# Non-contact human identification through radar signals using convolutional neural networks across multiple physiological scenarios

**DOI:** 10.3389/fdgth.2025.1637437

**Published:** 2025-10-07

**Authors:** Daniel Foronda-Pascual, Carmen Camara, Pedro Peris-Lopez

**Affiliations:** Department of Computer Science, Carlos III University of Madrid, Madrid, Spain

**Keywords:** contactless continuous identification, radar-based identification, heart dynamics, biometric authentication, different scenarios

## Abstract

**Introduction:**

In recent years, contactless identification methods have gained prominence in enhancing security and user convenience. Radar-based identification is emerging as a promising solution due to its ability to perform non-intrusive, seamless, and hygienic identification without physical contact or reliance on optical sensors. However, being a relatively new technology, research in this domain remains limited. This study investigates the feasibility of secure subject identification using heart dynamics acquired through a continuous wave radar. Unlike previous studies, our work explores identification across multiple physiological scenarios, representing, to the best of our knowledge, the first such exploration.

**Methods:**

We propose and compare two identification methods in a controlled Resting scenario: a traditional machine learning pipeline and a deep learning-based approach. The latter consists of using a Convolutional Neural Network (CNN) to extract features from scalograms, followed by a Support Vector Classifier (SVC) for final classification. We further assess the generalizability of the system in multiple scenarios, evaluating performance both when the physiological state is known and when it is not.

**Results:**

In the Resting scenario, the deep learning-based method outperformed the traditional pipeline, achieving 97.70% accuracy. When extending the identification task to various physiological scenarios, 82% of predictions exceeded scenario-specific confidence thresholds, achieving 98.6% accuracy within this high-confidence subset.

**Discussion:**

Our findings suggest that radar-based identification systems can match the performance of established biometric methods such as electrocardiography (ECG) or photoplethysmography (PPG), while offering the additional benefit of being contactless. This demonstrates the potential of radar heart signal analysis as a reliable and practical solution for secure human identification across diverse conditions.

## 1 Introduction

Some of the most prevalent and transformative advancements in the field of cybersecurity arise from the integration of biometric identification techniques. Biometric identification uses unique physiological and behavioral characteristics of individuals, offering a robust and multifaceted approach to identification, while simultaneously enhancing and streamlining the user experience. At the same time, these techniques hold relevance in the realm of modern healthcare, where the utilization of electronic health information plays a crucial role as a fundamental element.

Among various biometric techniques, Electrocardiogram (ECG or EKG) monitors the heart’s electrical activity, Electroencephalogram (EEG) measures brain activity, and Electromyography (EMG) captures muscle activity, enhancing biometric identification with diverse layers of uniqueness. Facial recognition, widely adopted, analyzes facial features, fingerprints with their distinct ridge patterns are already a standard in identification, and retina scanning examines eye blood vessel patterns enriching the spectrum of biometric identification techniques.

However, biometric identification methods that require contact, such as ECG and EEG, come with inherent drawbacks. Firstly, the need for specialized devices for signal acquisition can be inconvenient and limiting in terms of accessibility and portability. Additionally, direct physical contact with the user may lead to reluctance due to comfort or hygiene concerns or even provoke skin issues, for example, in the case of monitoring the heartbeat in premature babies [1]. Therefore, the ability to obtain cardiac motion without physical contact, particularly through radar technology, becomes highly compelling.

Furthermore, the integration of Doppler radar technology introduces a transformative dimension to non-contact biometric identification. Doppler radar, known for its efficacy in various applications such as weather forecasting [2] and physiological monitoring [3], extends its utility to the area of cybersecurity. By utilizing the Doppler principle, which detects subtle movements in the chest surface caused by heartbeat and respiration, radar technology enables non-intrusive cardiac motion detection. Doppler radar not only can ensure a secure and efficient identification process but also enhances user comfort by providing contactless means of capturing unique physiological characteristics. The versatility of Doppler radar positions it as a promising technology in advancing the capabilities of biometric identification systems, offering a compelling solution for the evolving landscape of digital security and opens up novel, user-friendly identification methods, such as heartbeat detection through Wi-Fi signals [4]. In the scientific literature on this topic, most studies on heart signal-based identification rely on contact-based techniques such as ECG. However, the field of identification using heart signals extracted without physical contact remains largely unexplored, with only a few studies employing non-contact methods like radar technology, which is the main motivation of this paper. In [5], a review of radar-based authentication methods is provided, where the majority rely on identifying individuals through their respiratory characteristics. In contrast, only five studies focus on cardiac signals, most of which were conducted on datasets with a relatively small number of subjects (4, 10, 11, 20, and 78 people, respectively). Therefore, studies in this field are still scarce and often performed on a limited number of subjects, mainly due to the shortage of available datasets. Moreover, most of these studies are carried out in laboratory settings with very stable conditions, which may differ from those in which this technology might eventually be applied. The aim of this article is twofold: first, to contribute to the study of this identification method by providing additional evidence of its viability and potential for good results using a dataset with 30 people; and second, to investigate its performance in scenarios where the subject is not necessarily in a resting position, thus assessing its applicability in more diverse situations—a novel aspect of this research.

In this case, data are collected with the assistance of medical experts in a laboratory setting. However, this approach does not need to be the only one. The technology could also be implemented in home environments, representing a significant area within the home health-care monitoring market [6]. For example, in the field of security, a potential application could be user authentication for logging into and maintaining an active session on a computer. A radar device installed on, for instance, the computer screen could capture the user’s cardiac signal while seated without requiring medical experts or physical contact. Additionally, the feasibility of obtaining cardiac signals from commodity Wi-Fi devices has been explored [4], and various portable systems and integrated radar chips have been demonstrated [3]. On the other hand, it is crucial to highlight that the sensitivity of medical data necessitates stringent security measures, which can complicate the deployment of various potential applications. However, this challenge is not exclusive to this technology; for example, other biometric methods, such as ECG, encounter similar issues but have still been successfully employed for real-time data collection and monitoring [7–9]. Moreover, several solutions, including encryption, have been proposed to safeguard this data. Given these precedents, it is reasonable to expect that the security measures for cardiac signals obtained via radar could follow analogous procedures, enabling their application in various contexts while ensuring the required level of security. On the other hand, the fact that this technique is contactless can facilitate the development of real-time applications.

Moreover, this study is based on radar-recorded cardiac signals from 30 healthy patients in a laboratory setting where random body movements were minimized. In a real-world environment, the presence of such movements would pose an additional challenge for the system’s applicability, similar to what happens with other biometric techniques like ECG or EMG, although there are currently no public datasets with these characteristics to study such effects. However, some studies have already begun investigating different methods to suppress these interferences and noise in the signal, thus enabling random body movement cancellation [10, 11].

The primary applications of this technology are likely in the field of security. Traditionally, many identification methods rely on tokens, such as passwords, which are vulnerable to theft. Other authentication systems utilize biometric data, including iris scans, fingerprints, or palm prints, and typically employ a one-time login process, with no subsequent security checks. In contrast, contactless identification supports a continuous and convenient identification process, enabling periodic verification of the user without causing disruption, thereby enhancing both security and usability. In clinical settings, this method could have the potential to substantially improve the collection and storage of patient medical data, including heart rate, respiratory rate, and blood pressure, for further analysis. By removing the need to manually enter patient identities into the system, this technology facilitates a more efficient and streamlined data collection process. Consequently, it leads to a more comprehensive and accurate database, thereby supporting better informed medical decisions and more effective patient management. The correct identification of patients in hospitals and healthcare centers is also critical in many cases, and an efficient and simple method to achieve this could help reduce errors in important processes such as the administration of medications [12]. Moreover, the trend in identification systems, as well as in the monitoring of physiological signals, is to eliminate physical contact in order to be as non-intrusive as possible, as is already the case, for example, with pacemakers, whose heart signals can now be read wirelessly.

The main contributions of this article can be summarized in three key points. First, we propose an efficient identification method based on radar sensing of cardiac motion in a resting scenario, a research area that has received little attention so far, and achieve an accuracy exceeding 97%. Second, we provide a comparative analysis between traditional machine learning and deep learning approaches for this task, demonstrating the clear advantage of deep learning methods and further examining their explainability. Finally, and most importantly, we advance beyond existing works by investigating subject identification across different scenarios in which the human body exhibits varied behaviors, thereby addressing the challenge of how identification models adapt to changes in physiological conditions.

The article is organized as follows. In the Section 2, we conduct a comprehensive review of existing literature pertaining to the detection of cardiac signals for person identification, with a specific focus on radar-detected cardiac signals. The Section 3 discusses the dataset employed for our study detailing the preprocessing steps and feature extraction methods applied. Moving on to the Section 4, we analyze the key findings derived from various experiments that were carried out. Finally, in Section 5, we draw some conclusions based on the obtained results.

## 2 Related work

In this section, we review some scientific literature relevant to our study, grounded in the domain of heart biometrics [13]. Within the field of user identification based on cardiac signals, the most extensively studied method is based on the ECG as analyzed in [14]. Remarkable results have been achieved, with evidence dating back to 2001 [15] demonstrating the efficacy of using a single-lead ECG for individual identification. A notable contribution in this domain is the work presented in [16], wherein ECG signals are transformed into scalograms. These scalograms are subsequently subjected to analysis using a CNN comprising seven convolutional layers, followed by classification using an SVC. The outcome of this approach yields an accuracy of 99.21%. Similarly, [17] reports comparable results by leveraging a heatmap derived from the ECG of multiple beats, referred to as Elektrokardiomatrix (EKM) as introduced in [18]. Employing a CNN with just a single convolutional layer on this heatmap achieves a high accuracy of up to 99.53% in a database comprising 18 individuals. Reference [19] employs a dual-path residual neural network alongside a split attention mechanism for ECG-based identification, achieving an accuracy of 99.6%. In [20] authors propose a 2-stage user identification system that integrates ECG signals with status information, addressing the challenges posed by signal variability due to physical and cognitive stress, achieving accuracies of up to 95.83%. More generally, ECG can be combined with other biosignals to achieve more comprehensive identification, as demonstrated in [21], where ECG and EMG signals are transformed into 2D spectrograms and analyzed using a multi-stream CNN, achieving an average accuracy of 96.8% in driver identification under various driving conditions.

While ECG captures variations in body surface potential, the Microwave Doppler sensor takes a different approach by attempting to extract heartbeat and individual feature quantities through time-frequency analysis without direct skin contact. The utilization of a 24-GHz microwave Doppler sensor is motivated by its capability to detect subtle chest surface vibrations induced by heartbeats. A critical challenge lies in the separation of signals associated with breathing and heartbeat. In various studies such as [22, 23] a Butterworth filter is used to extract the cardiac signal, eliminating the lower frequencies corresponding to respiration. However, in [24], the authors use Wavelet Packet Decomposition (WPD) to separate both signals, achieving errors less than 2% or 3.5% for respiration and heart rate, respectively, improving the accuracy of vital signals detection compared to Bandpass filter and Peak Detection. Subsequently, in [25], various methods are compared to determine which one extracts the cardiac signal better from the radar signal, among which Discrete Wavelet Transform (DWT) obtains the best results of all, including WPD. Moreover, the relationship between ECG and the cardiac signal extracted via radar has been previously explored, as in [26], where ECG signals are generated from cardiac activity detected using Doppler radar.

Within the area of cardiac signal detection, one of the initial objectives among researchers was to determine the heart rate using different techniques such as Fast Fourier Transform (FFT), Auto-Regressive Model (AR), or the detection of each single beat [27, 28]. But already in 2017, in [29], a method for identification based on the identification of the cardiac signal by a Continuous Wave (CW) radar was developed based on a fiduciary analysis of the cardiac signal. Fiduciary analysis refers to a method of signal processing or data interpretation that relies on distinctive fiducial points or features within the signal. These points serve as reference markers, aiding in the identification and extraction of specific information. On the other hand, non-fiduciary analysis involves alternative approaches that may not rely on specific fiducial points, often exploring broader characteristics or patterns within the signal for analysis. In this study, in order to avoid unwanted Random Body Movement (RBM), two radars are placed on each side of the patient. As a classifier, they use k-Nearest Neighbors (KNN) and SVC, obtaining a 98.61% balanced accuracy. In [30], instead of conducting a fiduciary analysis of the signal, the signal is segmented into individual heartbeats and resampled to a fixed number of samples. These samples are then fed into the classification algorithm. Each window is classified by the beats it contains through voting. However, the study is conducted on only a sample of 4 people. More recently, in [31], they transform the signal with Short Time Fourier Transform (STFT), creating spectrograms that are then classified with a Deep Convolutional Neural Network (DCNN).

Some of the current limitations of this technology include the still high price of the devices, although lower prices and greater availability are expected in the near future [32], and its sensitivity to RBM. Among the future challenges are capturing cardiac signals in multi-subject environments, enhancing the security of this data, and RBM cancellation [33]. In [1], for example, this latter point is investigated, where Non-negative Matrix Factorization is used to try to eliminate body movements in recordings of premature infants in the neonatal intensive care unit. In [34] a fiduciary identification method using radar is developed, focusing more on respiration than on the cardiac signal in order to perform subject identification in environments with more than one person. Other related areas being explored include the robustness to noisy bio-signals [35] or emotion recognition [36].

## 3 Materials and methods

### 3.1 Data

The study utilized a publicly available dataset provided by [37], collected by physicians at the University Hospital of Erlangen (Germany) from 30 healthy participants (14 males and 16 females) with an average age of 30.7 years. The radar system employed in the study had its focal point designed for a distance of around 40 cm from the region of interest (the thorax). It is based on Six-Port technology, designed for portable use. The measurements included five different physiological scenarios in which the patient may be during the recording:

• **Resting scenario:** Participants lay in a relaxed position for a minimum of 10 min. Calm breathing was instructed during this phase.
• **Valsalva maneuver scenario:** The Valsalva maneuver, involving forceful expiration against a closed glottis for 20 s, was performed three times with intervals of 5 min. Post-maneuver, the test person breathed out and resumed calm breathing.
• **Apnea scenario:** Participants held their breath in two defined states: inhaling completely before apnea and exhaling completely before apnea. Raw signals during the transition from normal respiration to apnea were recorded.
• **Tilt up scenario:** The tilt table was gradually raised to $70^{\circ }$ to trigger the Autonomic Nervous System (ANS) response. Hemodynamic changes, including significant alterations in blood pressure and heart rate, were anticipated.
• **Tilt down scenario:** Starting from the tilt up position with $70^{\circ }$ of inclination, the tilt table was lowered back to the starting position, and the recording continued for an additional 10 min. Similar ANS reactions were expected during the descent.

These scenarios, each serving a specific physiological purpose, were designed to investigate the impact on vital signs and autonomic functions during various physiological states. The duration of the recordings in the different scenarios may vary. In the Resting, Tilt Down, and Tilt Up scenarios, the recordings usually exceed 10 min. On the other hand, in Valsalva, they consistently exceed 15 min, while in Apnea, the duration typically ranges between 2 and 5 min. These differences in duration are due to the experimental design, which varies slightly for each scenario as described earlier.

Ethical and privacy considerations are crucial in research [38, 39]. The dataset used in this study was approved by the ethics committee of the Friedrich-Alexander-Universität Erlangen-Nürnberg (No. 85_15B). It is accessible at [40].

### 3.2 Signal preprocessing

From the recordings provided in the dataset, which include I/Q signals from the radar, the initial step involves decomposing these recordings into non-overlapping windows. Subsequently, ellipse fitting is applied to the I/Q point sets of each window following the method outlined in [41]. With these fitted ellipse parameters, arctan demodulation [42] is performed, yielding the signal corresponding to thoracic movement. An important aspect of preprocessing this type of signal compared to others like ECG is the potential increased presence of noise. Therefore, the study of its elimination becomes a crucial area to consider. In addition to system-related noise such as baseline wander, random body movements and chest displacement due to respiration must also be taken into account. In this case, from the demodulated signal, the cardiac signal is extracted using the Maximal Overlap Discrete Wavelet Transform method (MODWT), inspired by [25], where it has been demonstrated that this approach provides superior results for extracting the cardiac signal, at least for detecting peaks and heart rate. The Discrete Wavelet Transform (DWT) [43] is commonly employed to decompose a signal into distinct frequency components, facilitating a multi-resolution analysis while the MODWT [44] serves as an extension to the traditional DWT, introducing overlapping wavelet transforms to address specific limitations. Unlike the DWT, which decomposes a signal through successive non-overlapping segments, the MODWT utilizes overlapping segments in its decomposition process mitigating boundary effects that often occur in the standard DWT, particularly near the signal’s edges. In this study, the calculations employ the Morlet wavelet, similar to the methodology followed in [45]. However, instead of selecting just levels 4 and 5, we achieved better results by choosing levels from 1 to 5. After isolating the cardiac signal for each window, we proceed to decompose these signals into shorter-length frames, allowing for potential overlap between them. At this stage, the preprocessing varies based on whether traditional machine learning methods or a CNN will be used.

**Figure 1:**
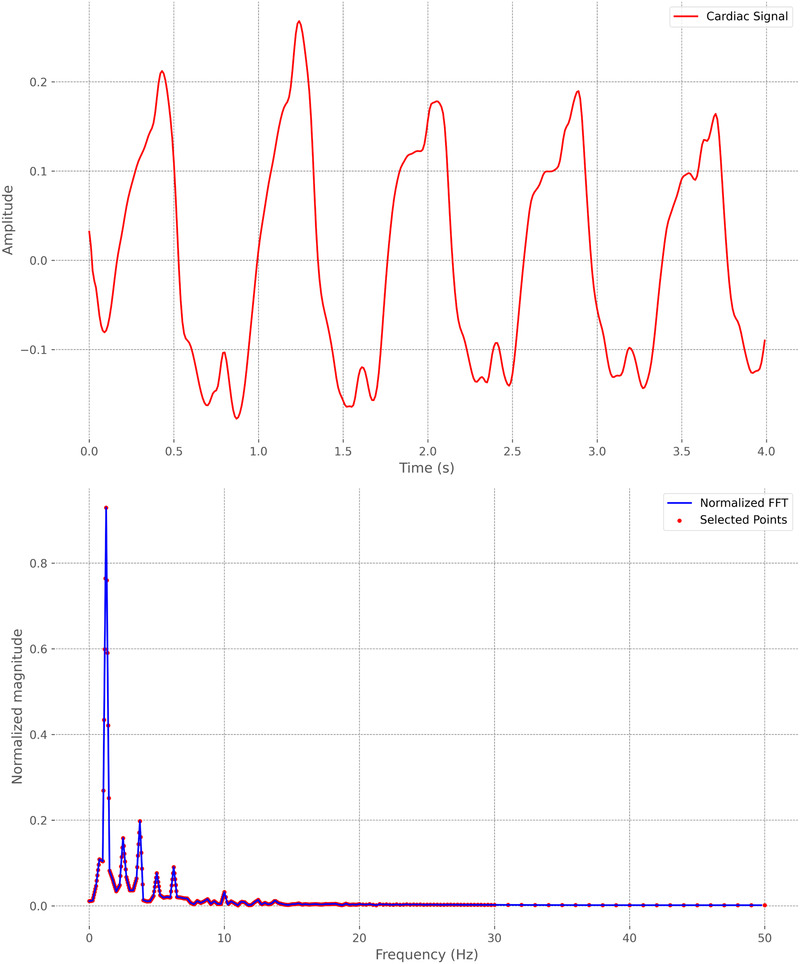
Example of a 4-second cardiac signal frame and its corresponding Fast Fourier Transform (FFT). Selected points on a fixed grid of 361 frequencies are highlighted.

In what we refer to as the “machine learning approach,” we use traditional machine learning methods that do not include deep learning, such as SVC, Random Forest, Extra-Trees, or Dense Neural Network (DNN). After segmenting the cardiac signal into frames, the Fast Fourier Transform (FFT) [46] is applied to the signal. A fixed grid of 361 points between 0 and 1 is established based on the frequency values derived from the FFT of the cardiac signals. This grid allows us to capture FFT values, resulting in a series of 361 points for each frame. In Figure 1, we can see an example of 4 s of a cardiac signal and its corresponding FFT, including the points that were selected as they belong to the fixed grid. Later with these points, to enhance subsequent efficiency, Principal Component Analysis (PCA) is applied for dimensionality reduction, reducing the data from 361 to 74 columns while explaining approximately 95% of their variance. The goal of using PCA is to reduce the dimensionality of the data and thus subsequently improve the performance of the different classification algorithms we will use, as studied in [47] and applied in [48–50]. With these 74 resulting points, which represent a cardiac signal frame corresponding to a patient, the objective is to use a classifier to determine which patient the frame corresponds to. Therefore, these 74 points will be the input to the classifier, while the output will be the class corresponding to the patient to whom the frame pertains.

**Figure 2:**
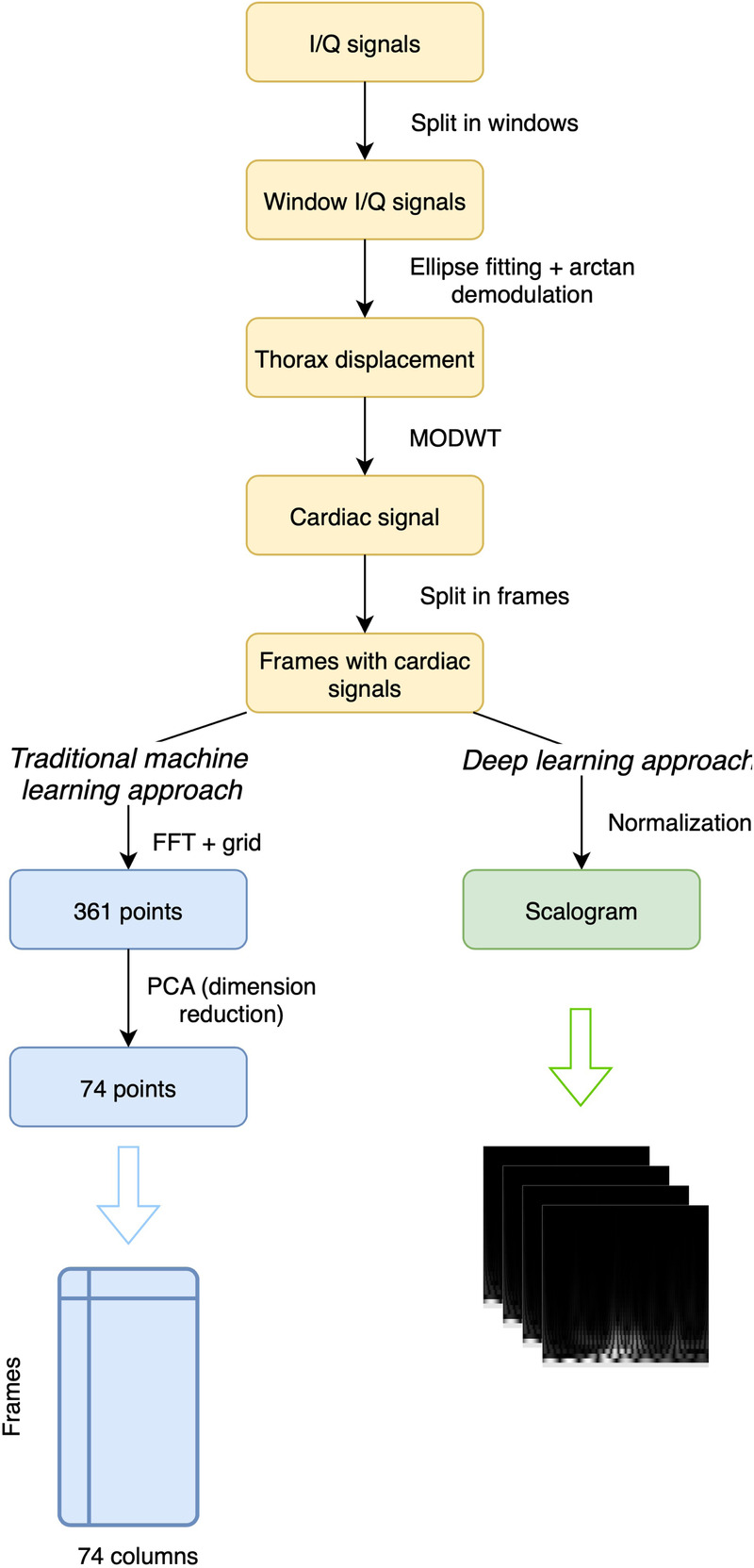
Preprocessing workflow for radar-derived I/Q signals.

In the deep learning approach, we use the normalized cardiac signal to generate a scalogram for each frame. The result after preprocessing is a scalogram, that visually summarizes the frequency content and time-varying characteristics of each frame. In addition to the scalogram, there are other signal-to-image conversion methods. In [51], the efficiency of several of them was compared for extracting signal features, such as Gramian Angular Field, Markov Transition Field, Recurrence Plot, Grey Scale Encoding, Spectrogram, and Scalogram, where in that case, the scalogram yielded the best results. However, the authors noted that the performance of each method can vary depending on the type of dataset used. While studies on cardiac radar signals are scarce, the scalogram has been extensively and successfully used in the field of ECG, as it performs well with signals sensitive to noise [52–56]. Specifically, the methodology followed in this study is very similar to [16], where ECG signals were converted to scalograms and subsequently classified using CNN and SVM. Given the successful outcomes that the scalogram has demonstrated in this field, we have chosen to apply this method in our study. The complete preprocessing workflow can be observed in Figure 2.

### 3.3 Train and test segmentation

Since this is a classification problem, the train/test split is not done by dividing the 30 subjects into two groups. Instead, we split each subject’s windows into training and testing sets, aiming to classify the test windows according to the patient they belong as accurately as possible. As we have conducted several different types of experiments (Sections 4.2–4.5), the division is slightly different in each case. For single-scenario splits, where the algorithm is trained using samples from the same scenario it is intended to predict, the last 25% of the windows in the recording has been selected as the test set, while the remaining windows (the first 75% of them) form the training set. The temporal split of the windows set appears to be a more appropriate approach, closely resembling what could be encountered in a real-world use case, as opposed to employing a random split of the windows. Naturally, with a random split, there is a possibility of having windows in the train set that are very similar to those in the test set, as they may be contiguous, thereby potentially improving results but deviating from reality applications. In practice, algorithms are expected to be trained on samples collected on specific dates, while the test set comprises samples, most likely, from subsequent days. Therefore, this temporal split seems more reasonable. In fact, it would be desirable to have recordings from different dates to allow for a more significant temporal separation between train and test samples, thus achieving a closer resemblance to the processes employed in practical applications of this kind of identification methods. In the case of trying to classify windows from unknown scenarios, one scenario is designated as the test set, and the others serve as the training set. It should be noted that, as the windows are non-overlapping, the risk of data leakage between partitions is effectively eliminated.

Another crucial aspect to consider is segmentation when performing cross-validation for hyper-parameter optimization (HPO). To achieve this, the training set must be divided into different splits. When dealing with a single scenario, this is accomplished by temporally dividing the windows of each patient. However, when working with multiple scenarios, two options have been considered: homogeneous cross-validation or heterogeneous cross-validation. In the former, windows from each scenario are divided into partitions, and then corresponding partitions from other scenarios are aggregated, ensuring that each fold contains windows from all scenarios. On the other hand, in heterogeneous cross-validation, splits are created without intermixing windows from different scenarios, with this latter option yielding superior results.

### 3.4 Feature extraction with CNN

**Figure 3:**
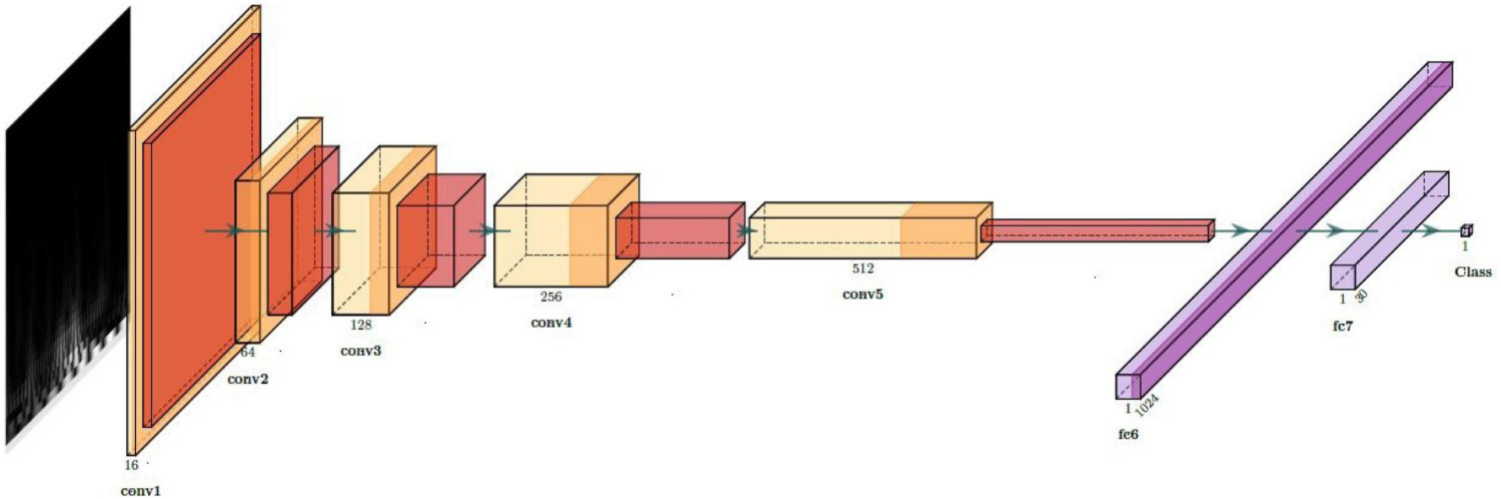
Architecture of the CNN used for feature extraction from scalograms.

Currently, CNNs are the predominant choice for feature extraction in the field of computer vision [57, 58]. The notable success achieved by CNNs in processing image data is attributed to their ability to extract crucial features from images, coupled with the computational prowess of Graphics Processing Units (GPUs) as processors. In our case, to extract features from the scalograms, we used a CNN composed of five convolutional layers followed by two fully connected layers. We applied batch normalization after each convolutional layer to normalize the input and mitigate internal covariate shift [59], and Rectified Linear Unit (ReLU) activation functions to introduce non-linearity after each batch normalization layer, facilitating the learning of complex patterns in the data. The fully connected layers consist of a linear layer with 1,024 output features followed by a dropout layer to mitigate overfitting [60]. The final linear layer produces the output logits, which are passed through a LogSoftmax activation for probability estimation during inference. This architecture is illustrated in Figure 3 and was implemented using the PyTorch framework. To train the network, the scalograms of each frame are provided as inputs in 200 × 200 pixel images, while the ground truth corresponds to the subject to whom that frame belongs, numerically coded.

After training, the final lineal layer is removed to obtain features, resulting in 1,024 features for each scalogram. In the CNN structure, convolutional layers are responsible for capturing hierarchical representations of the input scalograms, while the linear layers contribute to further refining these representations. The removal of the last classification layer ensures that the network functions as a feature extractor, providing a rich set of features that encapsulate the relevant information from the scalograms [61]. This feature representation is then fed into the subsequent SVC for classification.

The choice of this five-layer CNN is supported by prior studies in physiological signal classification. For example, [62] demonstrated that even moderate-depth CNNs can extract highly discriminative features for non-image physiological data when combined with PCA and SVM. Similarly, [63, 64] showed that features from scalogram-based CNNs effectively capture time–frequency information in real-world signals, significantly improving SVM classification performance. In [16], one-dimensional ECG signals were transformed into scalograms and classified with a seven-layer CNN, outperforming AlexNet and SqueezeNet as a deep feature extractor combined with SVM. Building on this work, we performed a comparative experiment with CNN architectures containing 3, 5, 7, and 9 convolutional layers to jointly evaluate performance and computational cost. The results, averaged over six repetitions, indicate that the five-layer CNN achieves the best trade-off between accuracy, number of trainable parameters, and inference time per sample. This model delivers the highest and most stable performance. Moreover, its inference time remains very low (0.29 ms per sample), which makes it well suited for real-time applications. For these reasons, we selected the five-layer CNN as the final architecture, as it balances model complexity and computational efficiency while preserving strong feature representation and classification performance.

### 3.5 Frame size optimization

Among the several hyper-parameters in consideration, two are particularly significant. Firstly, there is the selected temporal window width. As expected, in general terms, a larger window size will lead to higher accuracy in class predictions, as the algorithm has access to more data. Secondly, we divide each window into different frames that overlap. The size of these frames is a crucial hyper-parameter that requires optimization. Due to time constraints in computations, we employed the classical machine learning approach at this step.

**Figure 4:**
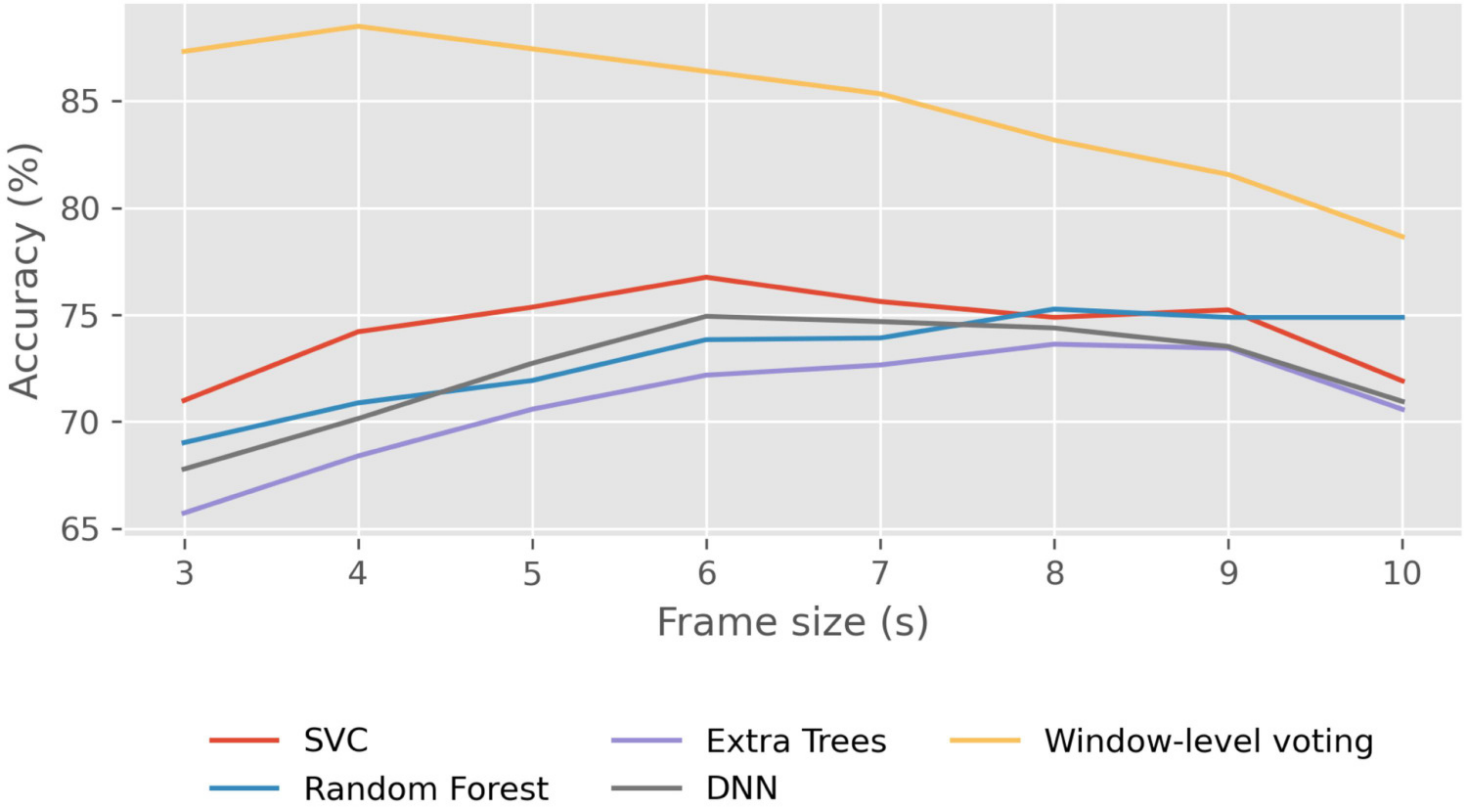
Effect of frame size on classification accuracy in the Resting scenario using traditional machine learning models. Average frame-level accuracy (%) is shown for SVC, Random Forest, Extra-Trees, and DNN, while the yellow line represents window-level accuracy obtained via soft voting across frames.

Setting an arbitrary window width of 12 s and a stride of 0.5 s between contiguous frames, we conducted the prediction process on the resting scenario with various frame sizes. The results are illustrated in Figure 4. In this figure, it is essential to note that the four models (SVC, Random Forest, Extra Trees, and DNN) are not predicting the class for each window but for each individual frame. Generally, we observe that, logically, when frames are very short, algorithms tend to make poorer predictions compared to longer frames, given the reduced amount of information. On the other hand, the yellow line represents the accuracy in window prediction obtained through soft voting with predictions for each frame from the four aforementioned models. While individual models may exhibit higher accuracy in predicting frames with longer duration, the predictions for windows are not as robust. Conversely, the highest accuracy in predicting window classes is achieved with 4 s frames. Thus, although the frames are somewhat shorter and consequently the model may not classify them as accurately individually, having more frames within each window results in more reliable predictions for the window class, leading to better overall results. For this reason, the frame size that we will use for the remainder of the study will be 4 s.

### 3.6 Explainability

To enhance the transparency and interpretability of our models, we analyzed their decision-making processes using explainability techniques. This approach aims to provide a deeper understanding of which features of cardiac signals are unique to each individual, enabling their identification. This study is framed within the domain of Explainable Artificial Intelligence (XAI) [65], which seeks to make machine learning models more interpretable without compromising their performance. XAI involves the development and application of methods that elucidate model behavior, bridging the gap between complex algorithms and human understanding.

We employed two techniques to interpret the outputs of our models: the Convolutional Block Attention Module (CBAM) and Shapley values. CBAM, first introduced in [66], is an attention mechanism that enhances CNNs by sequentially applying channel and spatial attention. The channel attention module identifies the most significant feature maps, while the spatial attention module highlights the most relevant regions within those feature maps. By focusing on these critical features and regions, CBAM provides an interpretable view of the model’s decision-making process, allowing us to better understand the patterns and characteristics captured by the CNN. CBAM has also been successfully applied in previous studies to explain models handling other types of biosignals, such as ECG [67–69]. To complement this, we used Shapley values to gain insights into the behavior of the model within the traditional machine learning approach. Shapley values [70], rooted in cooperative game theory, offer a robust method for explaining model predictions by quantifying the contribution of each feature to the output. In this context, features are treated as “players” in a coalition, and the prediction of the model is considered the “payout.” The Shapley value of a feature represents its average marginal contribution to the prediction, calculated over all possible subsets of features. This ensures a fair evaluation of each feature’s importance, accounting for its interactions with other features. The inclusion of these explainability techniques in this study substantiates the importance of specific features, ensuring that our methodologies remain transparent and rooted in domain-specific knowledge.

## 4 Results

Once we have completed the data preprocessing, we have each 4 s frame of cardiac signal from a specific subject synthesized into 74 values (traditional machine learning approach) or into a scalogram (deep learning approach). The goal of the different identification models we have tested is always to classify this information among the various patients in the study to determine which patient it corresponds to. In order to assess the model’s capability in user identification across the various scenarios available in the data, we conducted different experiments grouped into four sections. Firstly, we compared approaches using traditional machine learning and deep learning in a resting scenario and subsequently analyzed their results. In the “Identification in well-known scenario” section, we trained a model for each different scenario and studied its effectiveness in identifying patients in that specific scenario. On the other hand, in the “Identification in unknown scenario” section, we attempted to identify subjects in a scenario for which the model has not been trained, aiming to evaluate the model’s adaptability to new situations. Later, we trained a common model for all scenarios and attempted to identify patients with it. In addition, we conducted an open-set evaluation to investigate the system’s ability to reject previously unseen subjects, simulating real-world conditions where potential impostors may attempt to access the system. Finally, in the section “Feature Importance,” we present the results regarding the explainability of the models employed.

### 4.1 Performance metrics

In evaluating the effectiveness of each multi-class classification model, we employ a comprehensive set of metrics that collectively try to provide a good understanding of its performance across various dimensions. First of all, we must point out that in all scenarios the classes are balanced since the recordings of each one of the patients have approximately the same duration. Therefore, accuracy is the metric to which we pay the greatest attention, as it provides a fairly accurate insight into how the model is performing.

Given that we are addressing an identification problem, it is important to also consider both the False Acceptance Rate (FAR) and the False Rejection Rate (FRR). In our multiclass setting, these are computed on a one-vs-rest basis, making them directly comparable to precision and recall, respectively. FAR reflects the proportion of non-matching instances incorrectly accepted as a given class, while FRR captures the proportion of matching instances that are erroneously rejected.

Finally, to facilitate comparison with the results of other studies, we also provide precision, recall, and F1-Score. Precision is a metric that assesses the accuracy of positive predictions, representing the ratio of true positive predictions to the total number of instances predicted as positive. Recall, also known as Sensitivity or True Positive Rate, measures the model’s ability to identify all relevant instances, and the F1 score is a harmonic mean of precision and recall, providing a balanced assessment of the model’s performance. It is particularly useful when there is an uneven class distribution. We express all these metrics in percentages.

### 4.2 Identification in resting scenario

The first step that was attempted to identify patients has been solely based on samples taken in the resting scenario, which undoubtedly involves fewer complexities than the others. In this context, we possess recordings of approximately 10 min’ duration for each of the 30 patients. These recordings are divided into non-overlapping windows, with the last 25% of them allocated for testing and the initial 75% for training. The objective extends beyond assessing the feasibility of reliably identifying individuals through this method; it also seeks to compare the efficiency of traditional machine learning models against CNNs.

**Figure 5:**
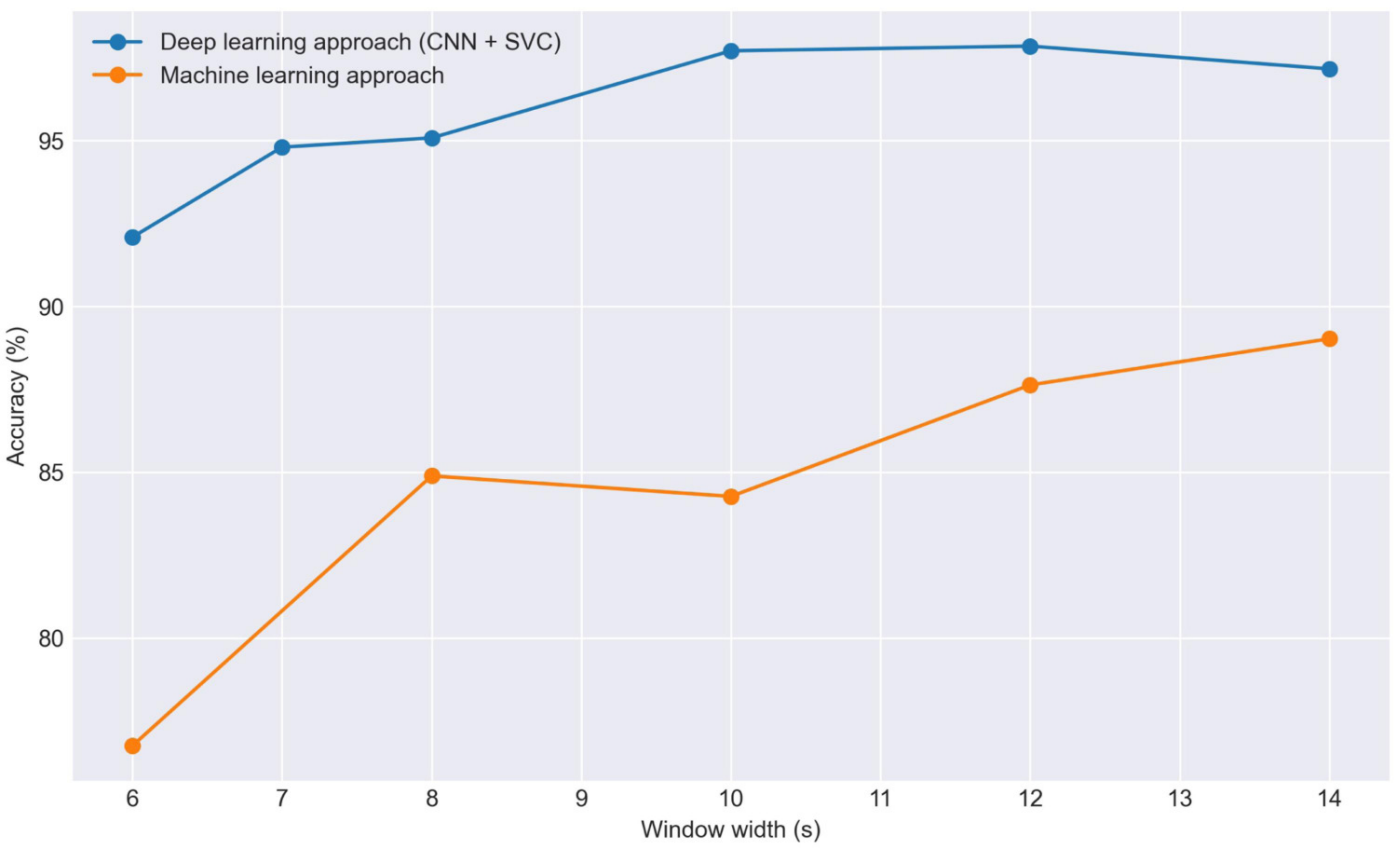
Effect of window size on classification accuracy in the Resting scenario comparing traditional machine learning models with the CNN+SVC approach.

In the machine learning approach, we tried several classification algorithms such as Support Vector Classifier (SVC), Random Forest (RF), Extra-Trees, Histogram-Based Gradient Boosting (HGB), K-Nearest Neighbors (KNN), Dense Neural Network (DNN), and X-Gradient Boosting (XGB). Notably, SVC, RF, Extra-Trees, and DNN demonstrate superior results. Subsequently, based on predictions from these four models, an assessment is made to determine if accuracy improves through the construction of a voting algorithm, incorporating both soft and hard voting. This approach has proven effective in enhancing results. After aggregating the probabilities for each frame in the test set from the four algorithms, we subsequently determine the predicted class for each window. As illustrated in Figure 4, the accuracy can experience a notable increase. Despite the aggregation of the four methods failing for certain frames, this shortcoming is attenuated by other frames within the same window. In Figure 5, we observe the effectiveness of this method based on the selected window width. In general terms and as expected, as the window width increases, the accuracy of the predictions also tends to increase. However, we can observe that the accuracy never reaches 90% with this method.

In the deep learning approach, we follow established methodologies for signal classification [16, 62–64], consisting of converting signals to scalograms, extracting features with a CNN, and classifying with an SVC. We used a five-layer CNN (Figure 3) to extract 1,024 features per scalogram by removing the final linear layer after training. Dimensionality reduction with PCA was then applied, reducing the features to 210 components while retaining approximately 95% of the variance, to accelerate SVC training. Lastly, we performed Hyper-parameter Optimization (HPO) on the SVC model and trained the classifier, which predicts the class of each frame. To determine the class of each window, a subsequent voting process is conducted with the predicted classes of each frame, both in soft and hard voting modalities. The results of both the traditional machine learning models and this latter approach are depicted in Figure 5.

As observed in the graph, the results obtained using CNN plus SVC are significantly superior reaching for 10 s windows 97.7% of accuracy. The specific outcomes for 10 s windows using both approaches are detailed in Table 1. Therefore, for the remainder of the study, we focus exclusively on this approach, discarding more conventional machine learning algorithms. Regarding the window size we will use, in general, a larger window size is anticipated to improve the model’s ability to identify the patient with greater reliability as more information is provided, which is consistent with our results using both approaches (Figure 5). Specifically, for the deep learning-based approach we observed an increase in accuracy as the window size extended from 6 to 10 s. Beyond the 10 s mark, however, the improvement in accuracy tends to plateau, suggesting that additional increases in window size yield diminishing returns. For this reason we have selected a 10 s window size as the standard for our study. Additionally, the window size most commonly used in the literature for biometric systems based on ECG or photoplethysmography (PPG) ranges from 3 to 10 s [14, 17, 71], being this result consistent with it.

**Table 1:** Classification metrics for 10 s windows in the Resting scenario for different approaches: Machine Learning (ML) and Deep Learning (DL), including a subset of DL predictions with confidence greater than Youden threshold.

Approach	Accuracy (%)	FAR (%)	FRR (%)	Precision (%)	Recall (%)	F1-score (%)
ML (All windows)	84.27	12.93	15.74	87.07	84.27	84.51
DL (All windows)	97.70	1.99	2.30	98.00	97.70	97.69
DL (Confidence > Youden thr.)	100.00	0.00	0.00	100.00	100.00	100.00

This model not only provides class predictions for each window but also quantifies the confidence of these predictions, which is particularly valuable when defining thresholds for decision-making in identification tasks. The predicted confidences were calibrated using Platt scaling [72] to mitigate the miscalibration inherent in the model’s raw outputs, thereby ensuring that the reported probabilities more faithfully represent the true likelihood of correct classification. The effectiveness of this calibration is evidenced by a reduction in the Expected Calibration Error (ECE) from 0.087 before calibration to 0.011 after calibration. An optimal decision threshold for this scenario was determined from the ROC curve using Youden’s index [73], yielding a value of 0.952. This threshold provides an objective criterion for distinguishing between correct and incorrect classifications, complementing the calibrated confidence values. Notably, 88.03% of the windows exceed the Youden threshold, achieving perfect accuracy with no false acceptances or rejections, as reported in the corresponding row of Table 1. These results underscore the reliability and practical applicability of the proposed approach in real-world scenarios.

Furthermore, to assess the statistical significance of these results, we compared the proposed CNN+SVC model with a benchmark baseline. The baseline was constructed by computing the Fourier transform of the radar-derived cardiac signals, extracting points on a uniform grid, applying PCA for dimensionality reduction, and classifying with a multilayer perceptron (MLP). McNemar’s test yielded a *p*-value < 0.001, confirming that the improvement achieved by the proposed model is statistically significant.

### 4.3 Identification in other well-known scenarios

In this section, we undertake a similar process to the one described previously, but this time applied to different scenarios present in the database. By training the model on the initial 75% of the patient recordings within a particular scenario, our objective is to subsequently identify the windows within the remaining 25% of that same scenario. Same as before, in order to enhance the experiment to closely simulate the potential deployment of the system in a real-world setting, we are temporarily splitting the recordings. This division serves to amplify the variability between both partitions, ultimately yielding more reliable metrics.

The results, presented in Table 2, are grouped by three configurations: *Random Split*, *Temporal Split*, and *Temporal Split* (*Confidence* > 90%), shown from left to right. Focusing on the *Temporal Split* columns, we observe that the Resting scenario yields the most accurate predictions. This is to be expected given that the resting scenario maintains homogeneity, unlike other scenarios where certain processes induce physiological alterations in the patient either throughout the entire recording or during specific segments. Secondly, the results for the Valsalva scenario are also favorable. This may be attributed to two reasons: firstly, this scenario encompasses recordings with notably longer duration thus, having more training data; secondly, the periods of disturbances (Valsalva maneuver) are relatively short, lasting 20 s, and thus have limited impact on the final outcome. Conversely, the least favorable results are observed in the Apnea scenario, potentially due to similar reasons but in the opposite direction. Recordings in the Apnea scenario are notably shorter than those in other scenarios, reducing the amount of data used to train the algorithm. Additionally, unlike the Valsalva scenario, the moments when the patient experiences apneas during the recording (in total, two apneas occur) may occupy a larger portion of the overall recording, significantly influencing the outcome. Furthermore, the apneas are not identical, occurring after inhalation and exhalation, respectively.

**Table 2:** Classification metrics (in %) for 10 s windows in well-known scenarios. Comparison between: Random Split, Temporal Split, and Temporal Split restricted to windows with prediction confidence greater than Youden threshold.

Scenario	Random split						Temporal split						Temporal split (Confidence > Youden thr.)					
	Acc.	FAR	FRR	Prec.	Rec.	F1	Acc.	FAR	FRR	Prec.	Rec.	F1	Acc.	FAR	FRR	Prec.	Rec.	F1
Apnea	79.13	17.73	23.75	78.69	76.25	74.40	65.57	23.84	43.16	72.99	56.84	57.79	93.65	4.91	14.29	86.03	85.71	84.31
Resting	99.77	0.26	0.24	99.74	99.76	99.74	97.70	1.99	2.30	98.00	97.70	97.69	100.00	0.00	0.00	100.00	100.00	100.00
Tilt Down	99.07	0.77	0.72	99.23	99.28	99.23	93.15	3.08	6.99	96.92	93.01	94.03	99.14	0.69	1.57	99.31	98.43	98.70
Tilt Up	96.06	3.77	3.63	96.23	96.37	96.15	84.55	14.33	15.14	85.67	84.86	84.28	97.23	3.14	3.69	96.86	96.31	96.27
Valsalva	99.86	0.19	0.12	99.81	99.88	99.84	96.18	3.51	3.91	96.49	96.09	96.14	100.00	0.00	0.00	100.00	100.00	100.00

**Figure 6:**
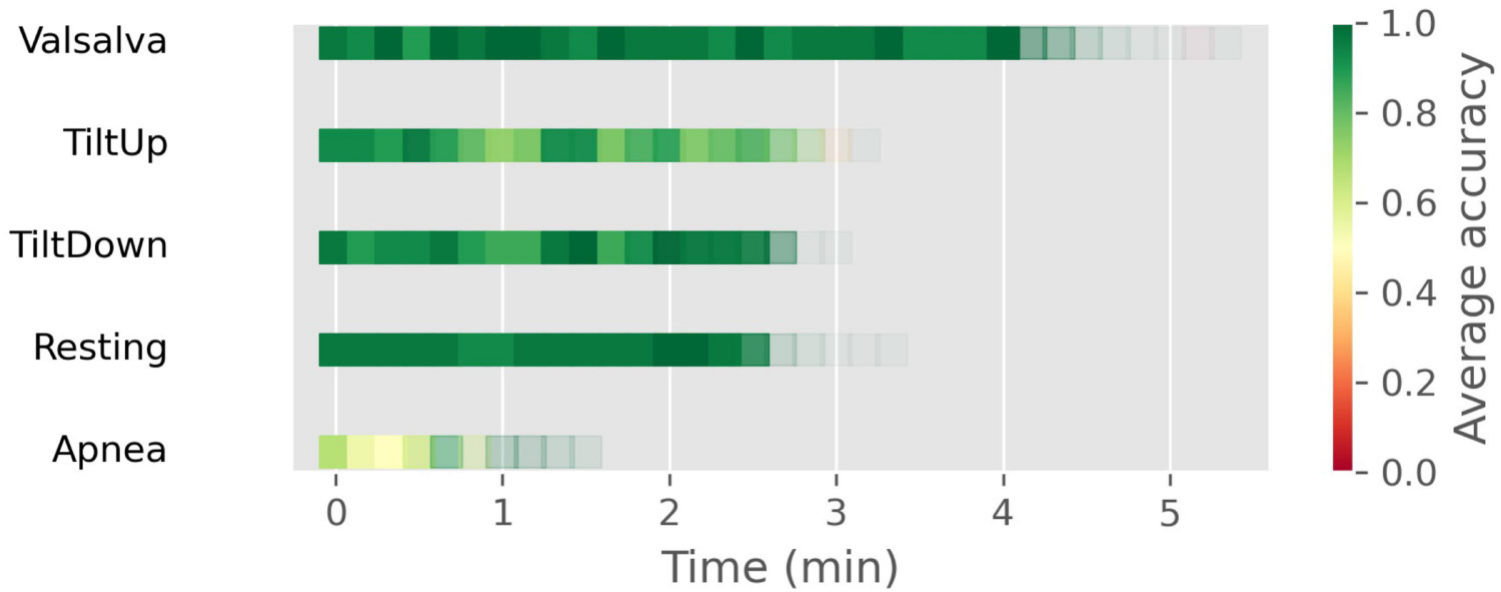
Temporal distribution of window-level classification accuracy across different well-known scenarios using the CNN+SVC approach. Each square represents a window in the test set, with color indicating average accuracy and transparency reflecting the number of windows at that temporal position.

If we analyze the results from a temporal perspective, Figure 6 is obtained, where windows located in the same temporal period of the test set for each scenario are depicted as squares. The color denotes the average accuracy in that temporal window while transparency reflects the quantity of windows existing at that moment (not all recordings have identical duration). Overall, accuracy remains consistent throughout the test set for each scenario. Perhaps only in Apnea, we can observe a concentration of worse results at the beginning of the test set. It’s possible that this is partly because those are the moments when the second apnea occurs.

As mentioned, the temporal split that we are using throughout this study provides greater similarity of experiments to a real-world scenario. This is because, in this approach, the test windows are temporally distant from the training set, resulting in comparatively lower accuracy. If we were to adopt a random split, dividing all windows into training and testing sets, as commonly done in various studies, we would likely achieve higher accuracies. However, these results might be somewhat artificial. This contrast is clearly observable in Table 2, where the random split (left section) shows notably better performance, albeit with potentially reduced reliability.

**Figure 7:**
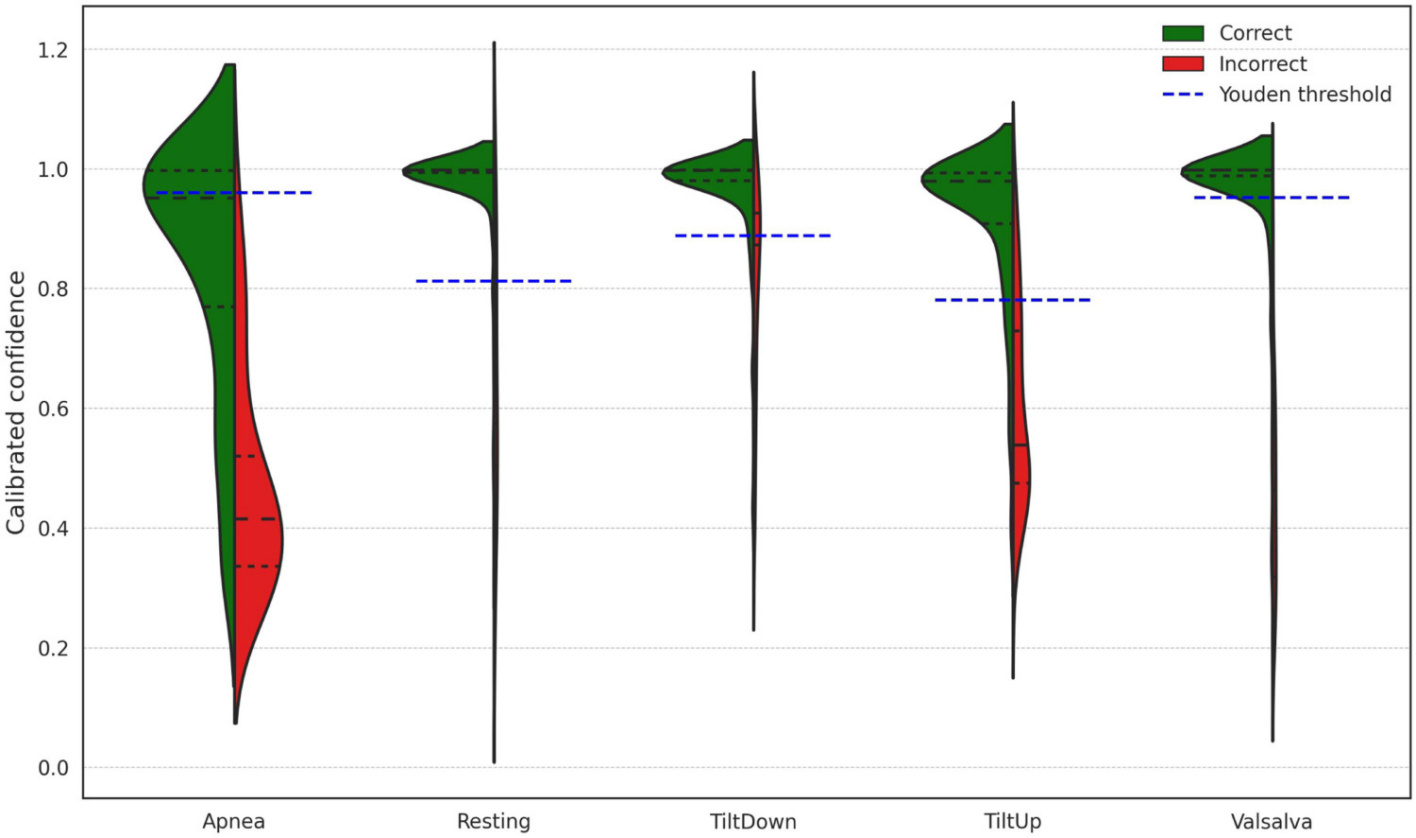
Distribution of window-level prediction confidences across different scenarios, indicating correct and incorrect predictions and the Youden threshold for each scenario.

Since the model provides a confidence level for each prediction, Figure 7 illustrates the distribution of windows according to prediction confidence and correctness, together with the Youden threshold computed for each scenario. Across all scenarios, 82.43% of the predictions exceed the Youden threshold for their respective scenario, achieving an accuracy of 98.59% within this subset. The rightmost section of Table 2 presents scenario-specific results for these high-confidence windows, confirming that focusing on predictions above the Youden thresholds substantially improves accuracy in all scenarios. These thresholds provide data-driven operating points derived from ROC analysis, offering an objective criterion for decision-making. To further evaluate the reliability of the model’s confidence estimates, Table 3 reports the Expected Calibration Error (ECE) before and after Platt scaling for each scenario, alongside the corresponding Youden thresholds. The results demonstrate that Platt scaling consistently reduces the ECE across all scenarios, indicating that the calibrated confidence values accurately reflect the true likelihood of correct classification.

**Table 3:** Calibration errors (ECE) before and after Platt scaling, and optimal thresholds (Youden index) for each test scenario.

Test scenario	ECE before calibration	ECE after calibration	Optimal threshold (Youden)
Apnea	0.159	0.084	0.781
Resting	0.087	0.011	0.952
Tilt Down	0.110	0.033	0.960
Tilt Up	0.160	0.041	0.813
Valsalva	0.118	0.026	0.889

As we mentioned, within this experiment, we assume that the scenario to which the window belongs is known. To enable the application of these results in a practical environment, it would be necessary to first classify to which scenario (or group of scenarios) a given window corresponds, something that has already been explored in fields such as human activity using Doppler radar [74], acoustics [75], or autonomous driving [76].

### 4.4 Identification in unknown scenarios

Given that the dataset has patient recordings in five different scenarios, one way to study the robustness of this type of identification is to attempt predictions in novel situations for the algorithm. The objective is to predict the class for each window within the test scenario without prior exposure to data from that specific scenario. To achieve this, we utilized all other scenarios as training data. The approach involved training a CNN on a random sample of windows from the training scenarios, subsequently we extracted features from all windows within those scenarios and trained a SVC on these features. The obtained results are presented in Table 4. For this part of the study, based on conclusions drawn from the resting scenario predictions we used 10 s wide windows and 4 s frames.

**Table 4:** Classification metrics for 10 s windows in unknown scenarios.

Test scenario	Accuracy (%)	FAR (%)	FRR (%)	Precision (%)	Recall (%)	F1-score (%)
Apnea	60.51	29.84	44.86	70.16	60.51	62.50
Resting	90.68	7.46	8.12	92.54	90.68	89.92
Tilt down	83.96	13.82	15.86	86.18	83.96	83.90
Tilt up	14.96	77.90	85.01	22.10	14.96	14.58
Valsalva	84.35	13.54	16.23	86.46	84.35	84.84

When interpreting the findings presented in this table, it is crucial to consider that the models made predictions for each scenario without prior training on that specific scenario, meaning they had not observed it before. It is not surprising that the most favorable results were achieved for the resting scenario, as instances of resting windows are inherent in scenarios such as Valsalva or Apnea. In these scenarios, there are intervals during the recordings where the patient is simply at rest, contributing to the algorithm’s familiarity with similar patterns in its training set.

The noteworthy performance for the Valsalva scenario can be attributed to this same fact. Each recording of this scenario includes three 20 s periods of executing the Valsalva maneuver, while the remainder of the time the patient is at rest. Consequently, a significant portion of this scenario aligns with the resting state, leveraging the model’s knowledge.

A significant divergence is observed in the results between Tilt Up and Tilt Down. It is important to note that in the Tilt Up scenario, the patient’s lying surface is elevated to $70^{\circ }$ and maintained for 10 min, whereas in Tilt Down, the surface returns to a horizontal position, and an additional 10 min are recorded. Unlike other scenarios recorded in a horizontal position, Tilt Up is unique in its almost vertical orientation. Furthermore, the body’s vital organs with the highest blood consumption are predominantly situated in the upper part. The transition from horizontal to vertical may likely induce a more pronounced alteration in cardiac effort compared to the reverse transition. These factors may contribute to the poorer results observed in the Tilt Up scenario, though other factors should not be ruled out. To further investigate this performance drop in the Tilt Up scenario, we analyzed several physiological indicators across the five scenarios. From the available dataset, which contains both ECG and blood pressure in addition to radar-derived cardiac signals, we extracted 30 random segments of 60 s from each subject and scenario, yielding a total of 4,048 samples. For each segment, we computed the mean heart rate (HR), the heart rate variability (HRV, measured as the root mean square of successive differences), and the mean blood pressure (BP). The analysis revealed that Tilt Up is indeed markedly different from the other scenarios: it exhibited a much higher mean HR (82.35 bpm, while all other scenarios ranged between 64 and 70 bpm), a substantially lower HRV (49.84, compared to values around 90–110 in the other scenarios), and the highest mean BP (92.19 mmHg, slightly above the remaining conditions). These differences provide quantitative evidence that Tilt Up constitutes a singular physiological condition compared to the other scenarios. This helps explain why the identification performance in this experimental setting is especially poor for Tilt Up, as the model trained on data from the remaining conditions is exposed to physiological patterns that differ significantly.

**Figure 8:**
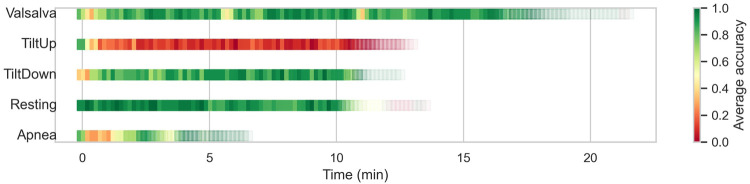
Temporal evolution of window-level prediction accuracy in unseen scenarios using CNN feature extraction and SVC classification. Each square represents a window in the test set, with color indicating average accuracy and transparency reflecting the number of windows at that temporal position.

Finally, given that several of the different scenarios are based on the execution of an experiment over time, we can analyze the prediction effectiveness of each window depending on the temporal moment, independently of the patient. In Figure 8, these results are presented, where we can observe, for example, how in the Valsalva scenario, windows related to the resting breaks between maneuvers are predicted accurately, while during the moments when the three maneuvers are typically executed, the prediction accuracy decreases. We have adjusted the transparency of each window based on the number of samples in that time frame. In the Tilt Up scenario, it is observed that at the beginning of the recording, when the platform has not yet started tilting, the algorithm is able to correctly identify the patient, whereas when the experiment begins, this capability is lost. Conversely, in the Tilt Down scenario, the first windows are predicted with lower accuracy, probably because the body is still influenced by the Tilt Up state, which is where the recording begins, while as it returns to the horizontal position, the predictions improve considerably. In the Resting scenario, accuracy is quite homogeneous, as expected in a scenario without alterations. Finally, in the Apnea scenario, especially in the windows corresponding to the first Apnea (after inhaling), predictions are not accurate.

Overall, these results indicate the model’s ability to identify the patient whenever the scenario is, if not the same, at least similar. As clearly observed, there is one scenario, Tilt Up, that is markedly different from the others, at least in terms of predictability by our model. However, others such as Tilt Down and Resting do not seem too dissimilar.

### 4.5 Common model for all scenarios

In previous experiments we developed individual models to predict each of the five distinct testing scenarios. Seeking to evaluate the efficiency of a model in recognizing subjects across diverse contexts, we extended our approach by training a unified model using data encompassing all scenarios. Subsequently, we assessed its capacity to identify the patient in any of the five scenarios. As noted earlier, there is a substantial imbalance in scenario durations, with the Apnea scenario being particularly underrepresented compared to the others. This imbalance can bias model training and reduce accuracy for shorter scenarios. To mitigate this, we implemented a synthetic up-sampling strategy targeting the Apnea recordings. Additional frames were generated by applying controlled augmentations to the existing cardiac signals, including slight Gaussian noise, temporal shifts, and amplitude scaling. These augmented signals were then converted into scalograms using the same wavelet and preprocessing parameters as the original frames, ensuring consistency across the dataset.

Similar to the previous approach, we employed a temporal split between training and testing data, using the first 75% of recordings for training and the last 25% for testing. The obtained results are disaggregated by test scenario, as shown in Table 5. In Table 6, we further compare accuracy across three different experiments: scenario-specific models for well-known scenarios, models applied to unknown scenarios, and the common model. As expected, identification performance in unknown scenarios is the least favorable. However, a more nuanced comparison emerges when contrasting the common model with individual scenario predictions. Accuracy remains approximately similar for four of the five scenarios, while a substantial improvement is observed in the Apnea scenario, likely due to the up-sampling strategy. Nonetheless, Apnea still remains the scenario with the lowest performance, followed by Tilt Up.

**Table 5:** Classification metrics for 10 s windows using a common model.

Scenario	Accuracy (%)	FAR (%)	FRR (%)	Precision (%)	Recall (%)	F1-score (%)
Apnea	80.67	20.86	25.32	79.14	71.70	73.38
Resting	97.52	2.37	2.44	97.63	97.56	97.52
TiltDown	94.95	5.04	5.09	94.96	94.91	94.76
TiltUp	83.45	17.19	16.39	82.81	83.61	82.28
Valsalva	96.16	3.76	3.97	96.24	96.03	96.04
Aggregated	92.75	7.05	7.30	92.95	92.70	92.75

**Table 6:** Accuracies for different scenarios and models.

Scenario	Well-known	Unknown	Common model
Apnea	65.57	60.51	80.67
Resting	97.70	90.68	97.52
Tilt down	93.15	83.96	94.95
Tilt up	84.55	14.96	83.45
Valsalva	96.18	84.35	96.16

**Figure 9:**
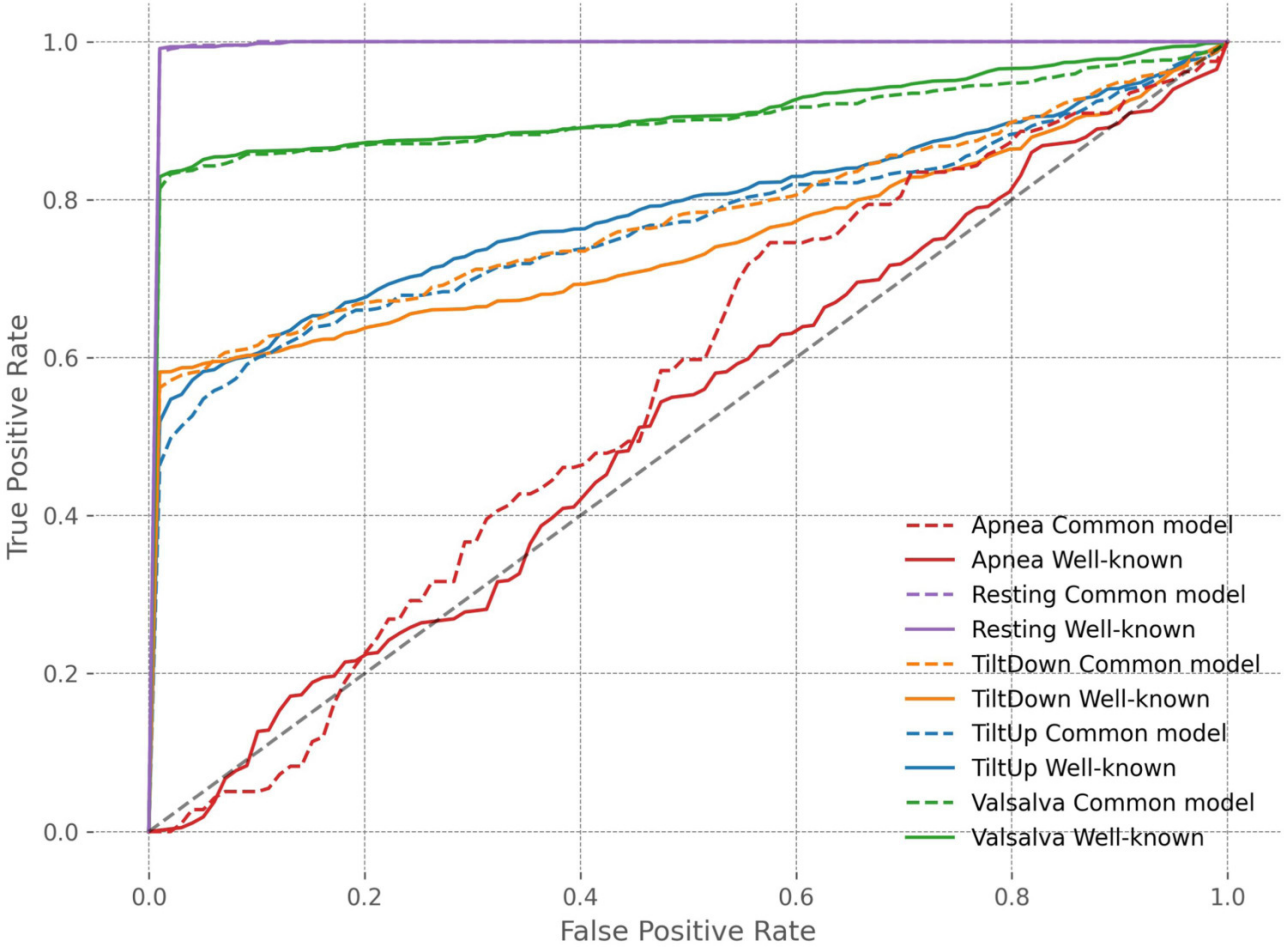
ROC curves across scenarios for well-known scenario and common model experiments.

To provide a comprehensive evaluation of biometric system performance, Table 7 reports additional metrics beyond accuracy, including Area Under the Curve (AUC) and Equal Error Rate (EER), both with 95% confidence intervals obtained via bootstrap methods. These metrics indicate that the common model maintains strong discriminative performance in straightforward scenarios such as Resting and Valsalva, with high AUC and low EER. In the underrepresented Apnea scenario, the common model benefits from knowledge transfer across scenarios, showing improved AUC and reduced EER, whereas in Tilt Up, performance slightly deteriorates, with lower AUC and higher EER, reflecting increased confusion between subjects across scenarios. Tilt Down exhibits intermediate behavior, with modest improvement under the common model. These trends are further illustrated in Figure 9, where ROC curves disaggregated by scenario provide a visual representation of the trade-off between true positive and false positive rates across all thresholds. The curves confirm that the common model can enhance identification in underrepresented scenarios such as Apnea, while performance in other scenarios such as Tilt Up may slightly deteriorate. Overall, these results highlight the potential of a unified model to leverage knowledge across scenarios, particularly for scarce data, while also revealing that model performance can vary depending on scenario-specific characteristics.

**Table 7:** Performance comparison between scenario-specific models and the common model across all scenarios, including Accuracy with 95% confidence intervals, Area Under the Curve (AUC), and Equal Error Rate (EER) with 95% confidence intervals.

Scenario	Well-known scenario					Common model				
	Accuracy	Acc. CI 95%	AUC	EER	EER CI 95%	Accuracy	Acc. CI 95%	AUC	EER	EER CI 95%
Apnea	65.57	57.37–73.76	0.517	0.503	0.429–0.546	80.67	72.88–87.16	0.554	0.477	0.398–0.524
Resting	97.70	95.94–99.27	0.999	0.007	0.000–0.011	97.52	95.95–98.88	0.999	0.009	0.001–0.010
TiltDown	93.15	90.64–95.21	0.749	0.245	0.231–0.259	94.95	92.69–96.82	0.778	0.219	0.207–0.237
TiltUp	84.55	81.08–87.90	0.788	0.226	0.211–0.248	83.45	79.97–86.63	0.766	0.258	0.235–0.275
Valsalva	96.18	94.79–97.48	0.914	0.095	0.084–0.102	96.16	94.65–97.50	0.906	0.102	0.091–0.110

### 4.6 Performance in open set conditions

In real-world applications, biometric systems often encounter subjects not present in the training set. To assess the robustness of our cardiac signal-based identification approach in such scenarios, we evaluated its performance under open-set conditions, where 5 individuals in the test set were completely unseen during training. These unseen subjects can be interpreted as adversaries attempting an impersonation attack, which requires the system to correctly reject impostors while accurately recognizing enrolled users. This evaluation allows quantifying the system’s ability to discriminate known subjects from unknown ones. Key metrics, such as the Equal Error Rate (EER) and associated Detection Error Trade-off (DET) curves, systematically characterize the trade-off between false acceptances and false rejections in this challenging context.

To implement this evaluation, we followed the experimental design of the well-known scenario described in Section 4.3. For each physiological scenario, a subset of five subjects was designated as unseen impostors, while the remaining subjects were used to train a CNN model. Using this CNN trained exclusively on the seen subjects of a given scenario, features were extracted for the training set, the scenario-specific test set, and the windows corresponding to the unseen subjects. An SVC classifier was then trained using the training set of seen subjects, and probability scores for each class were predicted for both the test set and the unseen subjects. To perform open-set evaluation, a one-vs-all strategy was applied, treating each seen subject as a positive class and all other subjects—including the unseen impostors—as negatives. The Equal Error Rate (EER) and Detection Error Trade-off (DET) curves were computed using all unseen subjects as impostors for each seen subject, and the EER was subsequently averaged across all seen subjects to provide a concise representation of open-set performance for each scenario.

**Figure 10:**
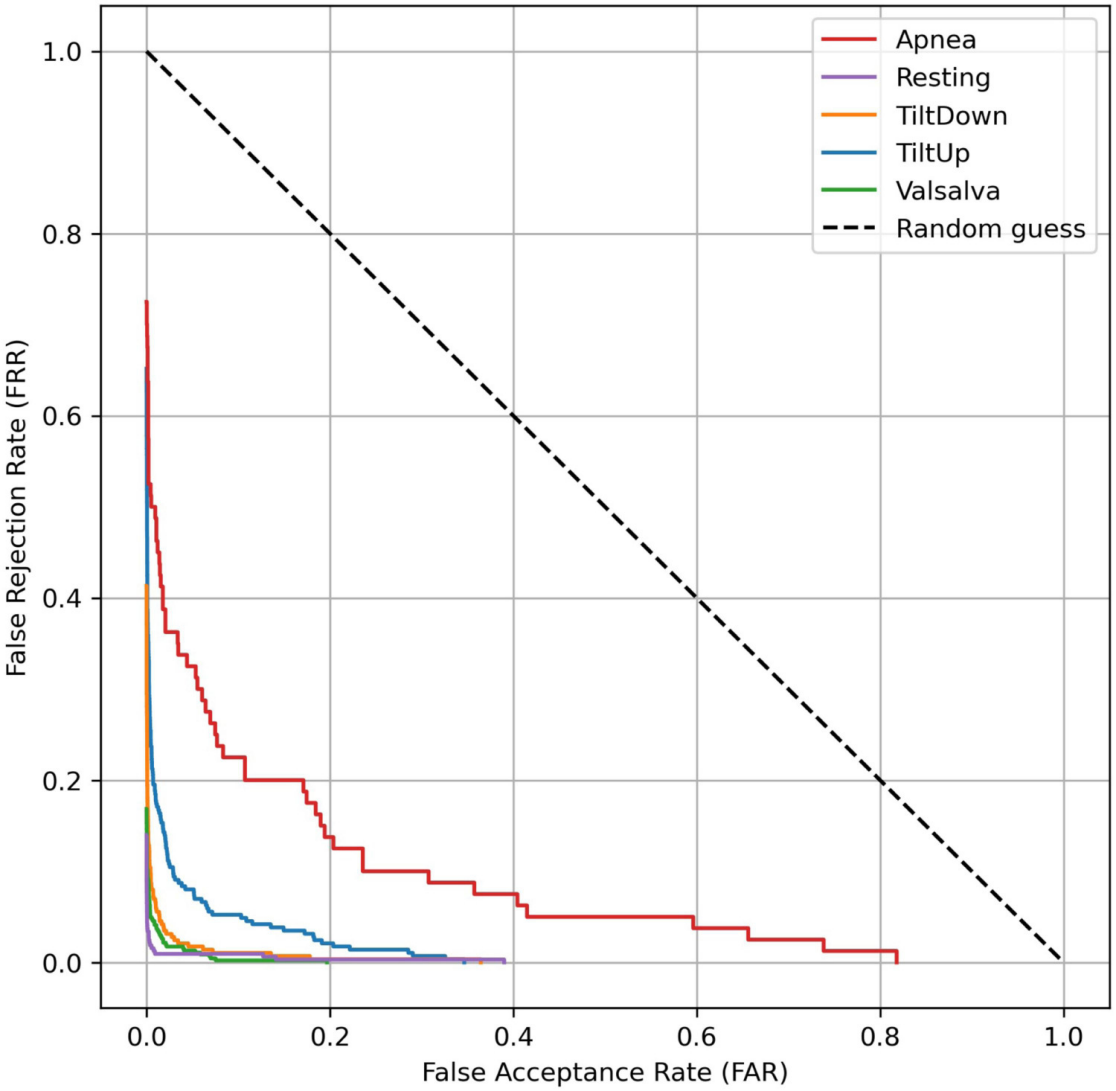
Detection Error Trade-off (DET) curves for open-set evaluation of cardiac signal-based identification. Each curve represents the average DET across all seen subjects within a physiological scenario, with unseen subjects treated as impostors.

The results of the open-set evaluation reveal a generally high robustness of the proposed identification system against unseen subjects. Specifically, the Tilt Down, Valsalva, and Resting scenarios exhibit very low EERs (1.45%, 1.26%, and 0.14%, respectively), indicating that the system reliably rejects impostors while correctly identifying enrolled users. The Tilt Up scenario shows a moderately higher EER of 5.63%, while the Apnea scenario presents the highest EER (19.15%), reflecting increased challenge in correctly discriminating users in this scenario. Overall, these findings confirm that the system maintains strong discrimination capabilities in an open-set context, with performance varying according to the physiological complexity of each scenario. The corresponding DET curves are illustrated in Figure 10, providing a visual representation of the trade-off between false acceptance and false rejection rates across different thresholds.

### 4.7 Feature importance

**Figure 11:**
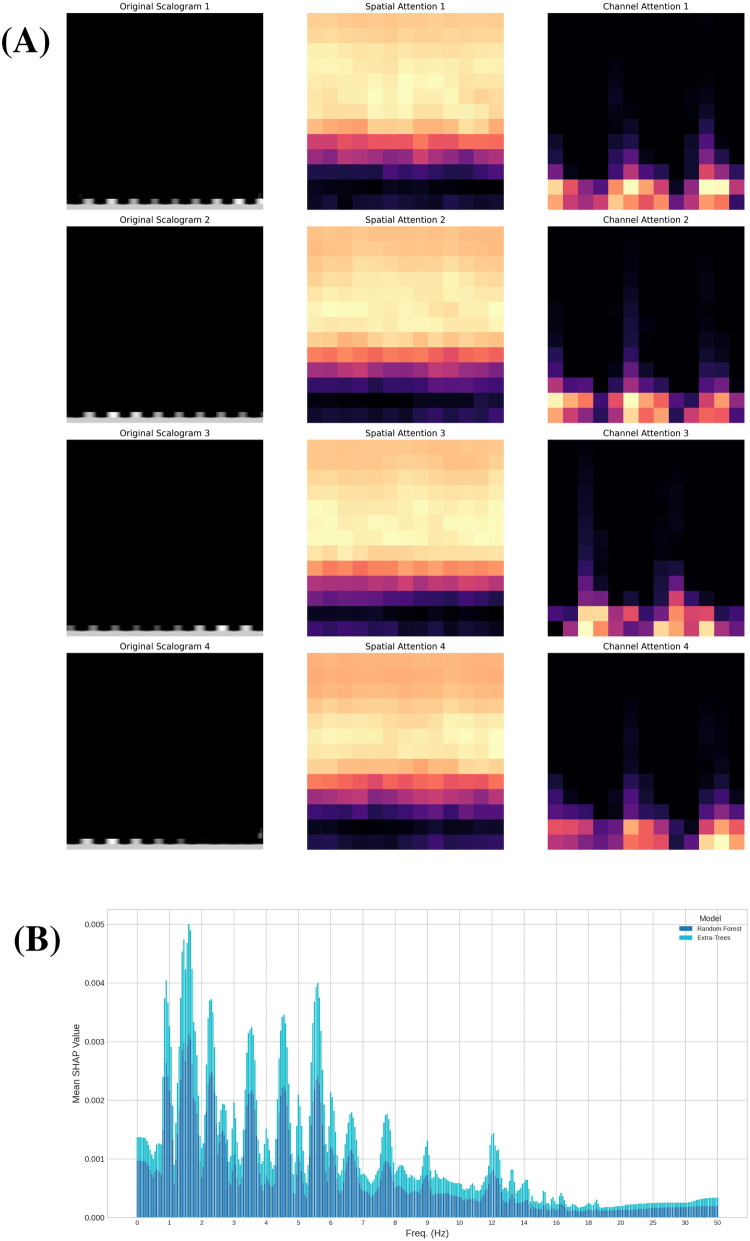
Explanation and attention analysis. **(A)** Attention maps: Randomly selected scalograms from the Resting scenario (left), corresponding spatial attention map (center), and channel with highest attention (right). **(B)** Shapley values for ML classifiers: Absolute mean of the Shapley values for the Random Forest and Extra-Trees classifiers in Resting scenario.

Building on the explainability framework described in Section 3.6, we leveraged the CBAM module to further investigate the specific regions and features that our deep learning model considers most significant when analyzing scalograms. The integration of CBAM, as detailed previously, provides a means to interpret the spatial and channel-level focus of the network. In our network, we incorporated the CBAM module following the activation function of the fourth convolutional layer in order to preserve sufficient resolution for identifying the regions of the scalograms where the network focuses its attention. After retraining the model for the Resting scenario, we obtained, for each scalogram, the specific regions where the network concentrates its attention and the feature maps that are most relevant. In Fig. 11A, we present a random sample of four scalograms (left) along with their corresponding spatial attention maps (center) and the feature map with the highest attention weight (right). In the central column of this figure, we observe that in all four cases, the network focuses particularly on the medium and high frequencies in the scalograms (horizontal axis in a scalogram represents time, while the vertical axis represents frequency). This could suggest that the most relevant information the model relies on to distinguish each subject is concentrated within these frequency ranges.

We further investigated feature importance by analyzing Shapley values within the traditional machine learning approach. This complementary analysis provides a detailed perspective on the contribution of individual features to the model’s predictions, enhancing our understanding of the decision-making processes behind certain ensemble classifiers. Specifically, we evaluated Shapley values using two of the four models in the ensemble: Random Forest and Extra-Trees, both of which led to similar conclusions. This analysis, conducted in the Resting scenario, provides a measure of feature importance for each of the 74 variables resulting from dimensionality reduction with PCA. Additionally, by applying the inverse of the projection matrix, we can assess the importance of each of the 361 frequencies extracted from the FFT of the cardiac signal (see Figure 1). This enables us to identify which frequencies have the greatest influence on predicting the subject associated with each signal. In Figure 11B, we present the absolute mean of the Shapley values of these frequencies for both classifiers. Both models highlight specific frequency ranges that significantly influence the predictions, with a particular focus on medium frequencies between 1 and 6 Hz. Notably, six frequency intervals within this range stand out, as emphasized in the figure. These intervals may represent the key frequency bands essential for distinguishing between subjects based on their cardiac signals.

In summary, these techniques have allowed us to gain insight into the factors that both models rely on to make their predictions. For the Random Forest and Extra-Trees classifiers, we identified specific frequency ranges that play a more significant role in determining the subject to which the sample belongs, all of which are located between 1 and 6 Hz. On the other hand, our CNN model demonstrates an internal focus on medium and high frequencies, which may indicate the presence of critical information necessary for accurate identification. The differences in the frequency ranges highlighted by each approach can be attributed to the distinct representations and methodologies employed: the traditional classifiers analyze features derived from the FFT, while the CNN focuses on patterns in the scalograms. These complementary insights reinforce the value of using diverse techniques to achieve a more comprehensive understanding of the problem.

## 5 Discussion & conclusions

In this study, we propose a contactless person identification method based on cardiac signal detection with a Continuous Wave radar and investigate its performance across different scenarios. The most promising approach in a Resting scenario involves extracting features from scalograms using a CNN and subsequently applying SVC for classification. This method outperforms traditional machine learning approaches, such as a voting classifier considering SVC, Random Forest, Extra-Trees, and Deep Neural Network. With Deep Learning, we achieve around a 10% improvement in prediction accuracy (see Figure 5) in the Resting scenario, reaching an accuracy exceeding 97% in a group of 30 individuals. Notably, this contactless and continuously authenticating method does not rely on fiducial point detection or analysis, aligning with findings in studies such as [77, 78], where superior results are reported for non-fiducial methods compared to fiducial-based approaches. The fact that there are no disturbances in this scenario and homogeneity is maintained throughout the recording allows for achieving good results, something that does not always occur in real-world cases.

In the literature, there are several proposals aiming to identify individuals based on cardiac signals. However, since it is a field in early development, there are not many studies yet that employ radar-acquired cardiac signals for this purpose. The most commonly used technique is based on ECG, while radar signals are often used to monitor and track subjects according to their position, movement and gait features. In Table 8 we can see some results in identification through cardiac signal extracted with radar compared to ours. Due to the scarcity of studies in this area, we have completed the table with some results from subject identification based on ECG.

**Table 8:** Comparative results of some radar-based and ECG-based identification proposals.

Year	Paper	Ref	Subjects	Results	Signal	Comments
2017	Cardiac scan: a non-contact and continuous heart-based user authentication system	[29]	78	BAC 98.61% EER 4.42%	Radar	Fiducial-based method
2017	Non-contact biometric identification and authentication using microwave doppler sensor	[28]	11	TAR 92.8% EER 3.9%	Radar	Time–frequency analysis with an auto-regressive model
2018	Contactless person identification using cardiac radar signals	[30]	4	Acc 94.6%	Radar	Classify individual heartbeats with SVM
2019	Heart ID: human identification based on radar micro-doppler signatures of the heart using deep learning	[31]	10	Acc higher than 80%	Radar	Short-time Fourier transform + DCNN
2022	Heart signatures: open-set person identification based on cardiac radar signals	[79]	30	Acc 99.17% (*with random split*)	Radar	Dipole deep learning model
2015	ECG based human identification using logspace grid analysis of second order difference plot	[88]	90	Acc 91.52%	ECG	
2017	HeartID: a multiresolution convolutional neural network for ECG-based biometric human identification in smart health applications	[89]	20–47	Acc 93.5%	ECG	
2020	Toward improving ECG biometric identification using cascaded convolutional neural networks	[90]	18–40	Acc 97.1%	ECG	
2020	An LSTM-based model for person identification using ECG signal	[91]	290	Acc 97.3%	ECG	
2022	ECGsound for human identification	[92]	18	Acc 96.6% FAR 0.2% FRR 0.4%	ECG	
2022	ELEKTRA: ELEKTRokardiomatrix application to biometric identification with convolutional neural networks	[17]	47	Acc 98.84% FAR 0.03% FRR 1.17%	ECG	
2022	BAED: a secured biometric authentication system using ECG signal based on deep learning techniques	[93]	90	Acc 99.49% FAR 0.14% FRR 0.99%	ECG	
2024	Our proposal in resting scenario		30	Acc 97.70% FAR 1.99% FRR 2.30%	Radar	CNN + SVC
2024	Our proposal in different scenarios		30	Acc 88.18% FAR 11.27% FRR 11.71%	Radar	CNN + SVC

BAC, balanced accuracy; EER, equal error rate; Acc, accuracy; TAR, true acceptance rate; FAR, false acceptance rate; FRR, false rejection rate.

When analyzing the results in the table, we must comment on two aspects that differentiate our study from previous ones. First, our approach differs from others in the temporal division of windows. In other studies, such as [79], the procedure followed is random splitting, which causes the test set to have windows very similar to those in the training set, as they can be contiguous. Our splitting methodology may slightly worsen results but brings the study closer to potential real-world applications. Using random splitting, we would achieve an accuracy of 99.77% in the Resting scenario (Table 2). Nevertheless, with temporal splitting, our results, surpassing 97% accuracy in Resting scenario, are not below the general level of other studies. More importantly, our model has been trained and tested with patients in different situations, something that previous studies do not consider and is likely the most significant novelty of this study.

We specifically evaluate the effectiveness of the identification process across different scenarios, considering both cases in which the scenario is known in advance and cases in which it is unknown. In the former, by applying an objective threshold based on the confidence of the predictions (e.g., the Youden threshold), highly secure identifications can be achieved, as illustrated in the rightmost columns of Table 2, which cover a substantial portion of the test cases. Concerning the experiment in single well-known scenario (see the Temporal Split column in Table 2), in three of them we achieve accuracies above 90%, while in Tilt Up and Apnea, the results are not as favorable. One factor that may be influencing the outcomes is the temporal design of the experiment in all scenarios but Resting. Since a physiological process takes place throughout the recording, and there is heterogeneity during this time, the test part is not exactly similar to the training part, making the algorithm’s task challenging. This is well evident in the Apnea scenario, in which only two apneas occur per patient, with the second one in the test set, leaving only one for training the algorithm. Additionally, this second apnea is different from the first as it occurs after exhaling. Nevertheless, this uncertainty of predictions is somehow reflected in the confidence provided by the model, as the results for those windows with confidence above the Youden threshold still remain satisfactory.

Additionally, we explore the method’s ability to predict patient identity in scenarios not previously encountered, a practical consideration given the variability in user physical conditions. While the outcomes are not unfavorable, they fall short of reaching good results. If accuracies in scenarios like Apnea or Valsalva are not low, it may be attributed to non-negligible periods of patient rest between maneuvers. Clearly, achieving robust results in a scenario necessitates prior training with patient data specific to that scenario, as exemplified in the Resting prediction section. Anyway, from these results we can also conclude the significant difference in the Tilt Up scenario compared to the others, probably for the reasons mentioned earlier. This scenario is the only one in which the body remains in an almost vertical position with vital organs with the highest blood consumption elevated above the heart, thus requiring a special effort and altering its behavior more significantly than in other scenarios.

Finally, we trained a model capable of identifying subjects in any scenario. Although the accuracy exceeds 90% in three out of the five scenarios, the results are still not sufficiently reliable for practical identification purposes. To address this, two potential enhancements can be considered. Firstly, by leveraging the confidence of model predictions, we can obtain robust results for the subset of samples in which predictions are deemed reliable. Additionally, another feature that could improve the predictive capability of the model in this multi-scenario context is incorporating a classifier that provides information about which scenario the sample belongs to before identifying the subject similarly to some studies like [80]. This way, the model’s accuracy could significantly improve.

In summary, using this technology in our dataset of 30 individuals, we can accurately identify a subject with very high precision when they are at rest (97.70% accuracy). This result is comparable to other biometric identification techniques, such as ECG (see Table 8), and is very close to the performance of more established methods like iris scans, facial recognition, or fingerprinting. Currently, with this dataset, the primary limitation in terms of reliability arises when identifying subjects in non-resting positions, where accuracy declines due to the increased complexity of these scenarios and potentially insufficient training data. However, by setting a confidence threshold for the model, we can achieve reliable identification in over 82% of cases (see Table 2), provided the scenario is known in advance. In this way, we approach accuracy levels comparable to more established biometric methods, while also leveraging the inherent advantages of this technology, such as not requiring light or physical contact, and enabling continuous authentication. Furthermore, the open-set evaluation demonstrates that the proposed method maintains strong resilience against impersonation attempts, with very low EER values in most scenarios (e.g., 1.26% in Valsalva and 0.14% in Resting), while only Tilt Up presents less favorable values. This confirms the system’s ability to reliably reject unseen impostors while recognizing enrolled users.

The explainability analysis conducted using a CBAM module revealed that our CNN model focuses primarily on medium and high frequencies in scalograms, suggesting that these regions contain critical information for subject identification. On the other hand, the analysis of Shapley values in traditional machine learning models (Random Forest and Extra-Trees) highlighted specific frequency ranges between 1 and 6 Hz, as the most influential for accurate predictions. These complementary approaches provide a deeper understanding of the decision-making processes and the key features leveraged by both deep learning and traditional models. Concerning the model’s complexity, we adopt a CNN architecture with 5 convolutional layers and two linear layers, inspired by [16] but with a slight reduction in complexity. A similar case is observed with the use of transfer learning to improve the network’s training, something that has already been studied with scalograms obtained from ECG signals [81]. However, its impact on the performance of the CNN in this case has not been explored and is left as future research. Furthermore, we address the critical aspect of system usability. In this case, a 10 s window of radar-detected signals is required to generate a scalogram, extract features with CNN, and classify using SVC. Once both models are trained, the runtime of the entire process is small, enabling seamless continuous identification.

Future research could explore additional strategies to further mitigate some of the limitations of the current dataset. In particular, the imbalance in scenario durations—especially the notably shorter Apnea recordings—may influence the performance of a common model due to the limited number of training samples. To address this, physiology-aware data augmentation techniques, such as those in [82], or deep learning-based augmentation methods like [83], could be investigated. Furthermore, exploring self-supervised contrastive learning (SSCL) could be an effective way to improve future models, as it can enhance model generalization and robustness by learning representations that are more invariant to subjects and postures, thereby facilitating identity discrimination even under previously unseen conditions [84].

A limitation of the results presented in this paper is the dataset’s small number of subjects, 30. As reflected in Table 8, this is a common issue across almost all previous studies in this field to date, with 30 patients representing a higher number than most of them. According to [85], the majority of datasets incorporating radar-based cardiac signals are considerably smaller, with a median of 12 subjects. The dataset used in this work is the second largest publicly available and uniquely includes data for each subject across five different scenarios. The only larger public dataset was excluded because it exclusively contains recordings from children under 13 years old. Therefore, this study represents a significant step forward in this emerging research area. Nevertheless, given this limitation, the present work focuses on the feasibility of this technique, without drawing definitive conclusions, as a much larger number of subjects would be required to do so, similar to what has occurred with other biometric techniques, such as ECG, which now has public databases containing hundreds of thousands of patients. Additionally, datasets with longer-term recordings collected across multiple sessions would be highly beneficial, enabling inter-session and longitudinal evaluation that better reflects realistic deployment conditions and supports robust assessment of system reliability over time.

After these results, the capability of this method based on the use of a CNN and an SVC to identify users from radar signals in various situations seems quite plausible, provided there is sufficient data to train the model. To draw more robust and generalizable conclusions, it would be beneficial to have recordings taken on different dates, varying health conditions, or under different external influences to verify that it is also possible to achieve identification across a broader spectrum of situations and over a wider time span. It would also be beneficial to assess the applicability of this technology in the real world by obtaining recordings of this type of signal in everyday settings rather than in a laboratory. Alternatively, this method can be integrated with other analogous identification methods utilizing radar signals, such as analyzing gait features [86] or spatial tracking [87], to enhance precision and robustness.

## Data Availability

The original contributions presented in the study are included in the article/Supplementary Material, further inquiries can be directed to the corresponding author.

## References

[1] Beltrão G Stutz R Hornberger F Martins WA Tatarinov D Alaee-Kerahroodi M. Contactless radar-based breathing monitoring of premature infants in the neonatal intensive care unit. Sci Rep. (2022) 12:5150. 10.1038/s41598-022-08836-3 35338172 PMC8956695

[2] Bringi V Chandrasekar V. Polarimetric Doppler Weather Radar: Principles and Applications. Cambridge, New York: Cambridge University Press (2001).

[3] Li C Lubecke VM Boric-Lubecke O Lin J. A review on recent advances in doppler radar sensors for noncontact healthcare monitoring. IEEE Trans Microw Theory Tech. (2013) 61:2046–60. 10.1109/TMTT.2013.2256924

[4] Shirakami I Sato T. Heart rate variability extraction using commodity wi-fi devices via time domain signal processing. In: *2021 IEEE EMBS International Conference on Biomedical and Health Informatics (BHI)*. (2021). p. 1–4.

[5] Islam SMM Borić-Lubecke O Zheng Y Lubecke VM. Radar-based non-contact continuous identity authentication. Remote Sens. (2020) 12:2279. 10.3390/rs12142279

[6] Islam SM Boric-Lubecke O Lubecke VM Moadi AK Fathy AE. Contactless radar-based sensors: recent advances in vital-signs monitoring of multiple subjects. IEEE Microw Mag. (2022) 23:47–60. 10.1109/MMM.2022.3140849

[7] Kiranyaz S Ince T Gabbouj M. Real-time patient-specific ECG classification by 1-D convolutional neural networks. IEEE Trans Biomed Eng. (2016) 63:664–75. 10.1109/TBME.2015.2468589 26285054

[8] Patro KK Prakash AJ Samantray S Pławiak J Tadeusiewicz R Pławiak P. A hybrid approach of a deep learning technique for real-time ecg beat detection. Int J Appl Math Comput Sci. (2022) 32:455–65. 10.34768/amcs-2022-0033

[9] Ribeiro HDM Arnold A Howard JP Shun-Shin MJ Zhang Y Francis DP. ECG-based real-time arrhythmia monitoring using quantized deep neural networks: a feasibility study. Comput Biol Med. (2022) 143:105249. 10.1016/j.compbiomed.2022.105249 35091363

[10] Zhu F Wang K Wu K. Doppler radar techniques for vital signs detection featuring noise cancellation. In: *2019 IEEE MTT-S International Microwave Biomedical Conference (IMBioC)*. (2019). Vol. 1. p. 1–4.

[11] Gouveia C Vieira J Pinho P. A review on methods for random motion detection and compensation in bio-radar systems. Sensors. (2019) 19:604. 10.3390/s19030604 30709017 PMC6387256

[12] Härkänen M Vehviläinen-Julkunen K Murrells T Rafferty AM Franklin BD. Medication administration errors and mortality: incidents reported in England and Wales between 2007–2016. Res Social Adm Pharm. (2019) 15:858–63. 10.1016/j.sapharm.2018.11.010 30528260

[13] Rathore AS Li Z Zhu W Jin Z Xu W. A survey on heart biometrics. ACM Comput Surv. (2020) 53:114. 10.1145/3410158

[14] Uwaechia AN Ramli DA. A comprehensive survey on ecg signals as new biometric modality for human authentication: recent advances and future challenges. IEEE Access. (2021) 9:97760–802. 10.1109/ACCESS.2021.3095248

[15] Biel L Pettersson O Philipson L Wide P. Ecg analysis: a new approach in human identification. IEEE Trans Instrum Meas. (2001) 50:808–12. 10.1109/19.930458

[16] Ozaltin O Yeniay O. A novel proposed CNN–SVM architecture for ECG scalograms classification. Soft Comput. (2023) 27:4639–58. 10.1007/s00500-022-07729-x 36536664 PMC9753894

[17] Fuster-Barcelo C Peris-Lopez P Camara C. ELEKTRA: ELEKTRokardiomatrix application to biometric identification with convolutional neural networks. Neurocomputing. (2022) 506:37–49. 10.1016/j.neucom.2022.07.059

[18] Li D Tian F Rengifo S Xu G Wang MM Borjigin J. Electrocardiomatrix: a new method for beat-by-beat visualization and inspection of cardiac signals. J Integr Cardiol. (2015) 1:124–8. 10.15761/JIC.1000133

[19] Sun L Li H Muhammad G. Randomized attention and dual-path system for electrocardiogram identity recognition. Eng Appl Artif Intell. (2024) 132:107883. 10.1016/j.engappai.2024.107883

[20] Kim Y Choi C. Utilization of a hierarchical electrocardiogram classification model for enhanced biometric identification. Comput Biol Med. (2025) 184:109254. 10.1016/j.compbiomed.2024.109254 39522129

[21] Choi G Ziyang G Wu J Esposito C Choi C. Multi-modal biometrics based implicit driver identification system using multi-tf images of ECG and EMG. Comput Biol Med. (2023) 159:106851. 10.1016/j.compbiomed.2023.106851 37099975

[22] Lopes ASD. Bio-radar applications for remote vital signs monitoring (Master’s thesis). Lisbon: Faculdade de Ciências e Tecnologia, Universidade Nova de Lisboa (2021).

[23] Cho HS Park YJ. Detection of heart rate through a wall using uwb impulse radar. Med Signal Process Biomed Clin Appl. (2018) 2018:4832605. 10.1155/2018/4832605 PMC5901946 29808110

[24] Liu L Xiao W Wu J Xiao S. Wavelet analysis based noncontact vital signal measurements using mm-wave radar. In: Yu Z Becker C Xing G, editors. Green, Pervasive, and Cloud Computing. Lecture Notes in Computer Science. Vol. 12398. Cham: Springer (2020). p. 3–14.

[25] Gouveia C Albuquerque D Pinho P Vieira J. Evaluation of heartbeat signal extraction methods using a 5.8 Ghz doppler radar system in a real application scenario. IEEE Sens J. (2022) 22:7979–89. 10.1109/JSEN.2022.3156474

[26] Chowdhury FA Hosain MK Bin Islam MS Hossain MS Basak P Mahmud S. Ecg waveform generation from radar signals: a deep learning perspective. Comput Biol Med. (2024) 176:108555. 10.1016/j.compbiomed.2024.108555 38749323

[27] Will C Shi K Schellenberger S Steigleder T Michler F Weigel R. Local pulse wave detection using continuous wave radar systems. IEEE J Electromagn RF Microw Med Biol. (2017) 1:81–9. 10.1109/JERM.2017.2766567

[28] Okano T Izumi S Kawaguchi H Yoshimoto M. Non-contact biometric identification and authentication using microwave doppler sensor. In: *2017 IEEE Biomedical Circuits and Systems Conference (BioCAS)*. (2017). p. 1–4.

[29] Lin F Song C Zhuang Y Xu W Li C Ren K. Cardiac scan: a non-contact and continuous heart-based user authentication system. In: *Proceedings of the 23rd Annual International Conference on Mobile Computing and Networking*. New York (NY, USA): Association for Computing Machinery (2017). MobiCom ’17. p. 315–28.

[30] Shi K Will C Weigel R Koelpin A. Contactless person identification using cardiac radar signals. In: *2018 IEEE International Instrumentation and Measurement Technology Conference (I2MTC)*. (2018). p. 1–6.

[31] Cao P Xia W Li Y. Heart ID: human identification based on radar micro-doppler signatures of the heart using deep learning. Remote Sens. (2019) 11:1220. 10.3390/rs11101220

[32] Saner H Knobel SEJ Schuetz N Nef T. Contact-free sensor signals as a new digital biomarker for cardiovascular disease: chances and challenges. Eur Heart J Digit Health. (2020) 1:30–9. 10.1093/ehjdh/ztaa006 36713967 PMC9707864

[33] Islam SMM. Radar-based remote physiological sensing: progress, challenges, and opportunities. Front Physiol. (2022) 13:955208. 10.3389/fphys.2022.955208 36304581 PMC9592800

[34] Islam SM. Non-contact and secure radar-based continuous identity authentication in multiple-subject environments (Ph.D. thesis). ProQuest Dissertations & Theses, Ann Arbor, MI, United States (2020).

[35] Jeon Y Kang SJ. Multi-slice nested recurrence plot (msnrp): a robust approach for person identification using daily ECG or PPG signals. Eng Appl Artif Intell. (2023) 126:106799. 10.1016/j.engappai.2023.106799

[36] Zhang Q Chen X Zhan Q Yang T Xia S. Respiration-based emotion recognition with deep learning. Comput Ind. (2017) 92–93:84–90. 10.1016/j.compind.2017.04.005

[37] Schellenberger S Shi K Steigleder T Malessa A Michler F Hameyer L. A dataset of clinically recorded radar vital signs with synchronised reference sensor signals. Sci Data. (2020) 7:291. 10.1038/s41597-020-00629-5 32901032 PMC7479598

[38] Li J. Open medical big data and open consent and their impact on privacy. In: *2017 IEEE International Congress on Big Data (BigData Congress)*. IEEE (2017). p. 511–4.

[39] Mocydlarz-Adamcewicz M Fundowicz M Galas-Świdurska D Skrobaa A Malicki J. Respecting patients’ privacy rights and medical data safety at a radiation oncology department during remote consultations and surveillance. Int J Radiat Oncol Biol Phys. (2024) 120:e562–3. 10.1016/j.ijrobp.2024.07.1246

[40] Schellenberger S Shi K Steigleder T Malessa A Michler F Hameyer L Data from: A dataset of clinically recorded radar vital signs with synchronised reference sensor signals. (2020). Available online at: https://figshare.com/articles/dataset/A_dataset_of_clinically_recorded_radar_vital_signs_with_synchronised_reference_sensor_signals/12186516 (Accessed December 3, 2023).

[41] Zakrzewski M Singh A Yavari E Gao X Boric-Lubecke O Vanhala J. Quadrature imbalance compensation with ellipse-fitting methods for microwave radar physiological sensing. IEEE Trans Microw Theory Tech. (2014) 62:1400–8. 10.1109/TMTT.2014.2321738

[42] Park BK Boric-Lubecke O Lubecke VM. Arctangent demodulation with dc offset compensation in quadrature doppler radar receiver systems. IEEE Trans Microw Theory Tech. (2007) 55:1073–9. 10.1109/TMTT.2007.895653 18002288

[43] Heil CE Walnut DF. Continuous and discrete wavelet transforms. SIAM Rev. (1989) 31:628–66. 10.1137/1031129

[44] Xiao F Lu T Wu M Ai Q. Maximal overlap discrete wavelet transform and deep learning for robust denoising and detection of power quality disturbance. IET Gen Transm Distrib. (2020) 14:140–7. 10.1049/iet-gtd.2019.1121

[45] Liu L Xiao W Wu J Xiao S. Wavelet analysis based noncontact vital signal measurements using mm-wave radar. In: Yu Z, Becker C, Xing G, editors. *Green, Pervasive, and Cloud Computing*. Cham: Springer International Publishing (2020). p. 3–14.

[46] Nussbaumer HJ Nussbaumer HJ. The Fast Fourier Transform. Berlin: Springer (1982).

[47] Choubey DK Kumar M Shukla V Tripathi S Dhandhania VK. Comparative analysis of classification methods with PCA and LDA for diabetes. Curr Diabetes Rev. (2020) 16:833–50. 10.2174/1573399816666200123124008 31971112

[48] Wisesty UN Lisnawati E Aditsania A Kusumo DS. Dimensionality reduction using principal component analysis for cancer detection based on microarray data classification. J Comput Sci. (2018) 14:1521–30. 10.3844/jcssp.2018.351.359

[49] Güler NF Koçer S. Classification of EMG signals using PCA and FFT. J Med Syst. (2005) 29:241–50. 10.1007/s10916-005-5184-7 16050079

[50] Uddin MP Mamun MA Hossain MA. Pca-based feature reduction for hyperspectral remote sensing image classification. IETE Tech Rev. (2021) 38:377–96. 10.1080/02564602.2020.1740615

[51] Garcia GR Michau G Ducoffe M Gupta JS Fink O. Time series to images: monitoring the condition of industrial assets with deep learning image processing algorithms. *arXiv* [Preprint]. *arXiv:2005.07031* (2020).

[52] Byeon YH Pan SB Kwak KC. Intelligent deep models based on scalograms of electrocardiogram signals for biometrics. Sensors. (2019) 19.935 10.3390/s19040935 30813332 PMC6412929

[53] Narin A. Detection of focal and non-focal epileptic seizure using continuous wavelet transform-based scalogram images and pre-trained deep neural networks. IRBM. (2022) 43:22–31. 10.1016/j.irbm.2020.11.002

[54] Benmalek E Elmhamdi J Jilbab A. ECG scalogram classification with CNN micro-architectures. Res Biomed Eng. (2022) 38:1–11. 10.1007/s42600-021-00188-7

[55] Kumar K Gupta K Sharma M Bajaj V Rajendra Acharya U. Insomnet: automated insomnia detection using scalogram and deep neural networks with ECG signals. Med Eng Phys. (2023) 119:104028. 10.1016/j.medengphy.2023.104028 37634906

[56] Mashrur FR Islam MS Saha DK Islam SR Moni MA. Scnn: Scalogram-based convolutional neural network to detect obstructive sleep apnea using single-lead electrocardiogram signals. Comput Biol Med. (2021) 134:104532. 10.1016/j.compbiomed.2021.104532 34102402

[57] Liu YH. Feature extraction and image recognition with convolutional neural networks. J Phys Conf Ser. (2018) 1087:062032. 10.1088/1742-6596/1087/6/062032

[58] Jogin M Divya GD Meghana RK Apoorva S. Feature extraction using convolution neural networks (CNN) and deep learning. In: *2018 3rd IEEE International Conference on Recent Trends in Electronics, Information & Communication Technology (RTEICT)*. (2018). p. 2319–23.

[59] Santurkar S Tsipras D Ilyas A Madry A. How does batch normalization help optimization? Adv Neural Inf Process Syst. (2018) 31:2488–98. Available online at: https://proceedings.neurips.cc/paper/2018/hash/905056c1ac1dad141560467e0a99e1cf-Abstract.html

[60] Srivastava N Hinton G Krizhevsky A Sutskever I Salakhutdinov R. Dropout: a simple way to prevent neural networks from overfitting. J Mach Learn Res. (2014) 15:1929–58. 10.5555/2627435.2670313

[61] Jogin M Mohana Madhulika MS Divya G Meghana RK Apoorva S. Feature extraction using convolution neural networks (CNN) and deep learning. In: *2018 3rd IEEE International Conference on Recent Trends in Electronics, Information & Communication Technology (RTEICT)*. IEEE (2018). p. 2319–23.

[62] Benkaddour MK Bounoua A. Feature extraction and classification using deep convolutional neural networks, PCA and SVC for face recognition. Traitement Signal. (2017) 34:77–91. 10.3166/ts.34.77-91

[63] Scarpiniti M Parisi R Lee YC. A scalogram-based cnn approach for audio classification in construction sites. Appl Sci. (2024) 14:90. 10.3390/app14010090

[64] Copiaco A Ritz C Fasciani S Abdulaziz N. Scalogram neural network activations with machine learning for domestic multi-channel audio classification. In: *2019 IEEE International Symposium on Signal Processing and Information Technology (ISSPIT)*. (2019). p. 1–6.

[65] Zednik C. Solving the black box problem: a normative framework for explainable artificial intelligence. Philos Technol. (2021) 34:265–88. 10.1007/s13347-019-00382-7

[66] Woo S Park J Lee JY Kweon IS. CBAM: convolutional block attention module. In: *Proceedings of the European Conference on Computer Vision (ECCV)*. (2018). p. 3–19.

[67] Ma K Chang’an AZ Yang F. Multi-classification of arrhythmias using resnet with cbam on CWGAN-GP augmented ECG gramian angular summation field. Biomed Signal Process Control. (2022) 77:103684. 10.1016/j.bspc.2022.103684

[68] Wang H Luo Z Yip JW Ye C Zhang M. ECGGAN: a framework for effective and interpretable electrocardiogram anomaly detection. In: *Proceedings of the 29th ACM SIGKDD Conference on Knowledge Discovery and Data Mining*. (2023). p. 5071–81.

[69] Fuster-Barceló C Guerrero-López A Camara C Peris-Lopez P. Exploring the power of photoplethysmogram matrix for atrial fibrillation detection with integrated explainability. Eng Appl Artif Intell. (2024) 133:108325. 10.1016/j.engappai.2024.108325

[70] Rodríguez-Pérez R Bajorath J. Interpretation of machine learning models using shapley values: application to compound potency and multi-target activity predictions. J Comput Aided Mol Des. (2020) 34:1013–26. 10.1007/s10822-020-00314-0 32361862 PMC7449951

[71] Shahid H Aymin A Remete AN Aziz S Khan MU. A survey on AI-based ECG, PPG, and PCG signals based biometric authentication system. In: *2021 International Conference on Computing, Electronic and Electrical Engineering (ICE Cube)*. (2021). p. 1–6.

[72] Platt JC. Probabilistic outputs for support vector machines and comparisons to regularized likelihood methods. Adv Large Margin Classifiers. (1999) 10:61–74. Available online at: https://www.researchgate.net/profile/John-Platt-2/publication/2594015_Probabilistic_Outputs_for_Support_Vector

[73] Youden WJ. Index for rating diagnostic tests. Cancer. (1950) 3:32–5. 10.1002/1097-0142(1950)3:1<32::AID-CNCR2820030106>3.0.CO;2-3 15405679

[74] Kim Y Ling H. Human activity classification based on micro-doppler signatures using a support vector machine. IEEE Trans Geosci Remote Sens. (2009) 47:1328–37. 10.1109/TGRS.2009.2012849

[75] Ma L Milner B Smith D. Acoustic environment classification. ACM Trans Speech Lang Process. (2006) 3:1–22. 10.1145/1149290.1149292

[76] Tang I Breckon TP. Automatic road environment classification. IEEE Trans Intell Transp Syst. (2011) 12:476–84. 10.1109/TITS.2010.2095499

[77] Odinaka I Lai PH Kaplan AD O’Sullivan JA Sirevaag EJ Rohrbaugh JW. Ecg biometric recognition: a comparative analysis. IEEE Trans Inf Forensics Secur. (2012) 7:1812–24. 10.1109/TIFS.2012.2215324

[78] Ingale M Cordeiro R Thentu S Park Y Karimian N. Ecg biometric authentication: a comparative analysis. IEEE Access. (2020) 8:117853–66. 10.1109/ACCESS.2020.3004464

[79] Yan B Zhang H Yao Y Liu C Jian P Wang P. Heart signatures: open-set person identification based on cardiac radar signals. Biomed Signal Process Control. (2022) 72:103306. 10.1016/j.bspc.2021.103306

[80] Koşar E Barshan B. A new CNN-LSTM architecture for activity recognition employing wearable motion sensor data: enabling diverse feature extraction. Eng Appl Artif Intell. (2023) 124:106529. 10.1016/j.engappai.2023.106529

[81] Gajendran MK Khan MZ Khattak MAK. ECG classification using deep transfer learning. In: *2021 4th International Conference on Information and Computer Technologies (ICICT)*. IEEE (2021). p. 1–5.

[82] Murshed RU Istiak MA Rahman MT Ashraf ZB Ullah MS Saquib M. A CNN based multifaceted signal processing framework for heart rate proctoring using millimeter wave radar ballistocardiography. Array. (2023) 20:100327. 10.1016/j.array.2023.100327

[83] Hatamian FN Ravikumar N Vesal S Kemeth FP Struck M Maier A. The effect of data augmentation on classification of atrial fibrillation in short single-lead ECG signals using deep neural networks. In: *ICASSP 2020-2020 IEEE International Conference on Acoustics, Speech and Signal Processing (ICASSP)*. IEEE (2020). p. 1264–8.

[84] Murshed RU Ullah MS Saquib M Win MZ. Self-supervised contrastive learning for 6G um-mimo Thz communications: improving robustness under imperfect CSI. *2024 IEEE International Conference on Communications Workshops (ICC Workshops)*. IEEE (2024). p. 220–6.

[85] Nocera A Senigagliesi L Raimondi M Ciattaglia G Gambi E. Machine learning in radar-based physiological signals sensing: a scoping review of the models, datasets and metrics. IEEE Access. (2024) 12:156082–117. 10.1109/ACCESS.2024.3482690

[86] Papanastasiou VS Trommel RP Harmanny RIA Yarovoy A. Deep learning-based identification of human gait by radar micro-doppler measurements. In: *2020 17th European Radar Conference (EuRAD)*. (2021). p. 49–52.

[87] Zhao P Lu CX Wang J Chen C Wang W Trigoni N. mID: Tracking and identifying people with millimeter wave radar. In: *2019 15th International Conference on Distributed Computing in Sensor Systems (DCOSS)*. (2019). p. 33–40.

[88] Altan G Kutlu Y. ECG based human identification using logspace grid analysis of second order difference plot. In: *2015 23nd Signal Processing and Communications Applications Conference (SIU)*. (2015). p. 1288–91.

[89] Zhang Q Zhou D Zeng X. Heartid: a multiresolution convolutional neural network for ECG-based biometric human identification in smart health applications. IEEE Access. (2017) 5:11805–16. 10.1109/ACCESS.2017.2707460

[90] Li Y Pang Y Wang K Li X. Toward improving ecg biometric identification using cascaded convolutional neural networks. Neurocomputing. (2020) 387:63–77. 10.1016/j.neucom.2020.01.006

[91] Jyotishi D Dandapat S. An lstm-based model for person identification using ECG signal. IEEE Sens Lett. (2020) 4:1–4. 10.1109/LSENS.2020.301265335582432

[92] Camara C Peris-Lopez P Safkhani M. ECGsound for human identification. Biomed Signal Process Control. (2022) 72:103335. 10.1016/j.bspc.2021.103335

[93] Allam JP Patro KK Hammad M Tadeusiewicz R Pławiak PB. BAED: a secured biometric authentication system using ECG signal based on deep learning techniques. Biocybern Biomed Eng. (2022) 42(4):1081–93. 10.1016/j.bbe.2022.08.004

